# Emerging Insights into Brain Inflammation: Stem-Cell-Based Approaches for Regenerative Medicine

**DOI:** 10.3390/ijms26073275

**Published:** 2025-04-01

**Authors:** Marie Karam, Alba Ortega-Gascó, Daniel Tornero

**Affiliations:** 1 Laboratory of Neural Stem Cells and Brain Damage, Department of Biomedical Sciences, Institute of Neurosciences, University of Barcelona, 08036 Barcelona, Spain; 2 Institut d’Investigacions Biomèdiques August Pi i Sunyer (IDIBAPS), 08036 Barcelona, Spain; 3 Centro de Investigación Biomédica en Red Sobre Enfermedades Neurodegenerativas (CIBERNED), 28029 Madrid, Spain

**Keywords:** border associated macrophages, Blood Brain Barrier, microglia, stem-cells

## Abstract

Neuroinflammation is a complex immune response triggered by brain injury or pathological stimuli, and is highly exacerbated in neurodegenerative diseases. It plays a dual role in the central nervous system, promoting repair in acute stages while aggravating disease progression by contributing to neuronal loss, synaptic dysfunction, and glial dysregulation in chronic phases. Inflammatory responses are mainly orchestrated by microglia and infiltrated monocytes, which, when dysregulated, not only harm existing neurons, but also impair the survival and differentiation of neural stem and progenitor cells in the affected brain regions. Modulating neuroinflammation is crucial for harnessing its protective functions while minimizing its detrimental effects. Current therapeutic strategies focus on fine-tuning inflammatory responses through pharmacological agents, bioactive molecules, and stem cell-based therapies. These approaches aim to restore immune homeostasis, support neuroprotection, and promote regeneration in various neurological disorders. However, animal models sometimes fail to reproduce human-specific inflammatory responses in the brain. In this context, stem-cell-derived models provide a powerful tool to study neuroinflammatory mechanisms in a patient-specific and physiologically relevant context. These models facilitate high-throughput screening, personalized medicine, and the development of targeted therapies while addressing the limitations of traditional animal models, paving the way for more targeted and effective treatments.

## 1. Introduction

The central nervous system (CNS) homeostasis and stable neuronal function are maintained optimally due to an essential physiological structure, the blood-brain barrier (BBB). With its selective permeability, active transport systems, and dynamic interactions with both CNS-resident and peripheral cells, the BBB plays a central role in brain function and pathology [1,2]. Disruption of this barrier can often lead to neurodegeneration, neuroinflammation, and other CNS pathologies.

BBB functions are essentially dictated by the specific architecture of its cellular components, their dynamics, and their continuous crosstalk. The BBB is made up of a neurovascular unit (NVU) consisting of several key cell types. Endothelial cells (EC), connected by tight and adherens junctions, limit vascular permeability. Mural cells, including pericytes and smooth muscle cells (SMC), help maintain barrier integrity and regulate blood and cerebrospinal fluid (CSF) flow. Perivascular macrophages (PVM) support immune surveillance, drainage, and barrier maintenance. Finally, glial cells (astrocytic endfeet forming the glia limitans) and neurons regulate BBB function by facilitating communication and ensuring neurovascular coupling [3,4]. These components work in concert to orchestrate and sustain a wide range of brain mechanisms critical to CNS health. The BBB plays an important role in both health and disease, and its disruption can lead to neuroinflammation, a major hallmark of numerous neurological and neurodegenerative disorders [5].

Neuroinflammation is the brain’s immune response to injury, infection, or pathological conditions, and it mainly involves the activation of glial cells, including microglia, astrocytes and the CNS’s resident border-associated macrophages (BAM), including PVMs [6,7]. While acute neuroinflammation serves as a protective mechanism to eliminate pathogens and damaged cells, chronic or dysregulated neuroinflammation is at the basis of neurodegenerative diseases such as Alzheimer’s disease (AD), Parkinson’s disease (PD), stroke, and multiple sclerosis (MS) [7]. This balance between protective and deleterious effects underlines the importance of deepening our knowledge in understanding the pathways that regulate neuroinflammatory processes.

Interestingly, the BBB and neuroinflammation are intricately connected and can affect one another. A healthy BBB plays active roles in regulating the CNS immune response by modulating microglial activation, T cell infiltration, and the release of pro- and anti-inflammatory mediators [7]. On the other hand, chronic inflammation can impair BBB integrity by disrupting tight junctions and increasing permeability, allowing peripheral immune cells and potentially harmful substances to infiltrate the brain [8]. This results in an increase in the brain’s inflammation load, leading to worsened and perpetuated BBB degeneration. For this, neuroinflammation plays a central role in the onset and progression of neurodegenerative diseases, creating a harmful feedback loop where each process can drive and amplify the other.

In the early stages of neurodegeneration, the chronic activation of glial cells and BAMs releases pro-inflammatory molecules such as cytokines (e.g., Tumor necrosis factor alpha (TNF-α), interleukin-1-beta (IL-1β)) and reactive oxygen species (ROS). This creates a toxic environment that promotes oxidative stress and encourages the additional buildup of toxic solutes like amyloid beta (Aβ) and tau proteins, resulting in synapse disruption, neuronal death, and accelerating disease progression. As neurons are damaged, they release molecules called damage-associated molecular patterns (DAMP), which further activate glial cells, sustaining the inflammatory response [9]. Therefore, misfolded proteins like Aβ or α-synuclein (α-syn) act as ongoing triggers for inflammation, worsening the cycle [10]. Breaking this cycle by targeting inflammation early on or addressing its effects, such as protein buildup, could help delay or halt the spread of neurodegenerative diseases.

Efforts to overcome neuronal death are currently being centered around stem cell therapies. However, understanding the interplay between the BBB, its cellular components, and neuroinflammation is crucial for developing these kinds of therapeutic strategies. Preserving BBB integrity and modulating inflammatory responses are key to helping transplanted stem cells survive and do their job. These factors are essential to prevent and mitigate neurodegenerative diseases and to protect cognitive function.

## 2. Microglia and BAMs’ Role in Neurological Disorders

**Figure 1:**
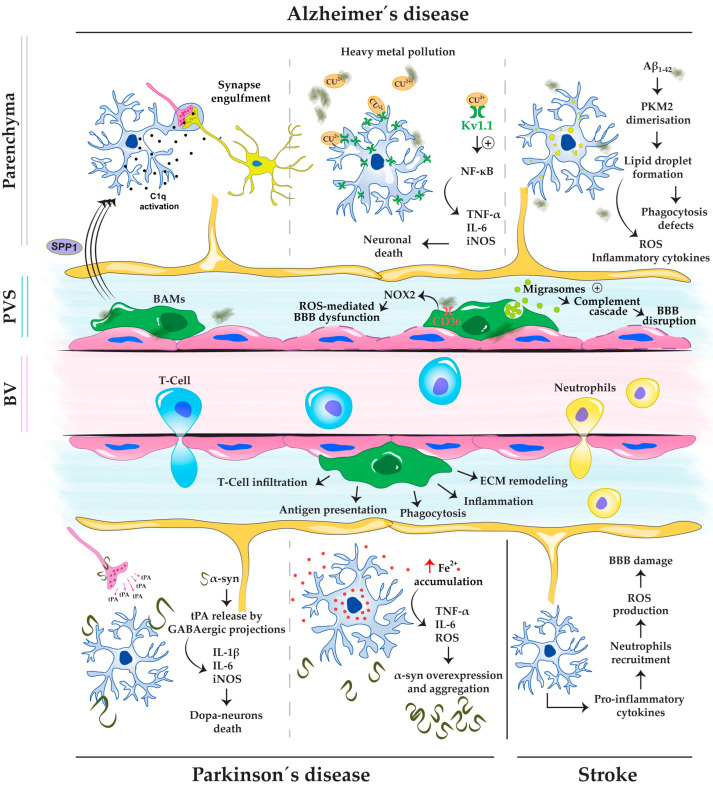
Scheme summarizing the innate response of microglia and BAMs in AD, PD, and stroke. While studies on microglia in neuroinflammatory responses have been extensive, research on BAMs remains less explored. Further research into BAMs is essential for expanding our understanding of their contributions to neuroinflammation and developing targeted therapeutic strategies for these diseases. BAM response is common for PD and stroke.

The brain’s immune system consists of both innate and adaptive components, each playing distinct yet interconnected roles in maintaining neural homeostasis and responding to injury or infection. Innate immunity in the brain is primarily mediated by microglia, the resident immune cells that continuously survey the environment for signs of damage or pathogens. Microglia are the first responders that are activated quickly upon injury to release cytokines and chemokines that recruit peripheral immune cells to the site of injury [11] (scheme in Figure 1). They also play a key role in clearing debris and dead cells through phagocytosis [12]. PVMs, newly characterized BAMs, also contribute to innate immune responses by releasing inflammatory mediators and maintaining the integrity of the BBB [6] (scheme in Figure 1).

In contrast, adaptive immunity in the brain is more tightly regulated by the BBB, which limits the entry of peripheral immune cells. However, under pathological conditions where the BBB is deteriorated, T cells and B cells can infiltrate the brain and either exacerbate inflammation or promote tissue repair depending on their phenotype [9,13,14]. The interactions between microglia/PVMs and adaptive immune cells are critical for regulating the balance between neuroprotection and neurodegeneration [9]. By modulating this crosstalk to promote anti-inflammatory and tissue-regenerative responses, innovative therapeutic approaches can be envisioned for addressing conditions marked by chronic neuroinflammation.

### 2.1. Microglia

In pathological conditions and upon activation, microglia shift from a resting, ramified shape to an amoeboid macrophage like-form [15], accompanied by changes in surface receptor expression, increased ROS production, and the release of pro-inflammatory chemokines and cytokines. While some secreted factors can be neurotrophic and protective, most contribute to inflammation, oxidative stress and neurotoxicity [15]. Activated microglia also phagocytose, present antigens, proliferate, and recruit additional microglia to amplify inflammation.

Interestingly, microglial activation exhibits a diverse array of phenotypes, which can generally be classified into pro-inflammatory and anti-inflammatory states. However, this binary classification fails to capture the considerable overlap and continuous spectrum between these states. Reactive microglia in particular are highly dynamic in their role as inflammatory mediators. They possess a variety of pathways that allow them to transition across the spectrum from pro-inflammatory to anti-inflammatory phenotypes. While these states are often simplified into the classical M1 and M2 subtypes, this dichotomy has increasingly been called into question and is now considered overly simplistic [16].

Moreover, an in-silico study based on integrating single-nucleus and single-cell RNA sequencing datasets from healthy and pathological human brain samples, has succeeded in creating a Human Microglia Atlas (HuMicA). HuMicA found that microglia activation exhibits a dynamic and complex transcriptional state with multiple specialized subtypes existing simultaneously, rather than progressing linearly from a “resting (homeostatic)” state to an “activated’’ one in response to stimuli [15]. Despite microglia’s complex heterogeneity and activation states, this study showed that microglial responses to neuroinflammation was not disease specific, and no clear tendencies specific to one pathology were actually observed [15]. However, it is important to note that in AD, different microglia subsets, such as populations expressing genes associated with lysosome, fatty acid beta-oxidation, lipid metabolism, lipoproteins and others associated with the ribosomes complex, have been identified to be involved in this disease [15]. More and more, microglia activation and pro-inflammatory states are being reported to occur way before the AD plaque formation. This positions microglia as a first effector for neurodegenerative disease progression and also as the first therapeutic target to modulate its effects.

#### 2.1.1. Alzheimer’s Disease

AD is the predominant type of neurodegenerative disorder and the leading cause of dementia [17]. It is characterized by the deposition of extracellular Aβ plaques, neurofibrillary tangles, the loss of synapses and neurons, as well as gliosis [17]. Among these pathological features, synapse loss in the frontal cortex has been shown to correlate with the disease initiation and progression more effectively than plaque formation/counts and neuronal loss [18,19]. Interestingly, in an AD mice model, microglial-mediated synaptic pruning was found activated in preplaque brains. This synapse loss was induced by synaptotoxic oligomeric Aβ (oAβ) and C1Q binding to synapses and activating the complement cascade in microglia. Therefore, microglial activation following this cascade, and the resulting synapse loss, suggests that inflammatory pathways can act as early mediators of synapse loss and dysfunction that occur in AD models before plaques form [20]. This microglial involvement underscores the role of inflammatory pathways as early mediators of neurological dysfunction in AD.

In addition to Aβ, dysregulated metal ion homeostasis—particularly copper (Cu(II))—has been shown to contribute to microglia-mediated inflammation. Cu(II) ions dysregulated reuptake and increased concentration in the synaptic cleft, has been shown to directly bind to Aβ, promote Aβ aggregation and cause oxidative damage responsible for neuronal loss and cognitive decline [21,22,23]. Strikingly, excessive extracellular copper also induces neurotoxic microglial activation in a yet-to-be-discovered way [21,24]. Therefore, a recent study has tackled this question and found that Cu(II) exposure is responsible for upregulating the expression of the voltage-gated potassium channel Kv1.1 found on microglia. Cu(II) and Kv1.1 binding triggered the downstream activation of the PI3K/Akt-ERK-NF-κB pathway, which promotes and mediates microglial activation and the pro-inflammatory state [21].

On that matter, other pathways beyond NF-κB have also been identified as key regulators of inflammatory responses. Notably, Neuraminidase 1 (NEU1) plays a crucial role in microglial activation. Its deficiency can independently activate the Akt and NFAT1 pathways, driving pro-inflammatory microglial activity characterized by the release of TNF-α and Chemokine (C-C motif) ligand 3 (CCL3), thereby contributing to AD independently from the NF-κB pathway [25].

A similar phenomenon is observed with lipid droplets (lipid-storing organelles), which are sites of inflammatory cytokines production and therefore a hallmark for inflammation and stress response in myeloid cells. In AD, microglia shows lipid droplet accumulation that is associated with phagocytosis defects, increased ROS and inflammatory cytokines secretion. This accumulation was shown to be mediated by pyruvate kinase M2 (PKM2) dimerization. PKM2 inhibition following capsaicin treatment diminished inflammation, neuronal loss, memory impairment and tau pathology, offering a therapeutic approach for AD [26].

#### 2.1.2. Parkinson Disease

Parkinson’s disease (PD) ranks as the second most common neurodegenerative disorder and the primary progressive motor condition globally [27]. It is characterized by α-syn accumulation, the loss of dopaminergic neurons in the substantia nigra (SN) pars compacta, leading to a significant reduction in dopamine levels in the striatum [28]. This dopamine deficiency manifests as the hallmark motor symptoms of PD, including bradykinesia, resting tremor, rigidity and postural instability [27,28].

Microglia are showing to be early mediators in PD. An increase in activated microglia accompanied by proinflammatory molecules (IL-1β, IL-6, iNOS and cyclooxygenase 2 (COX2)) was reported as early as 1 month of age, well before cell death occurs in a PD transgenic mice line [29]. This was due to the direct activation of the microglial inflammatory response by mutated α-syn in a dose dependent way, indicating a possible early-event participation of microglia in PD pathogenesis [29]. As previously mentioned, microglia are highly heterogeneous with diverse states following their environmental cues. They can shift from protective to harmful states depending on disease progression. In chronic models of PD, microglia depletion has shown neuroprotective effects such as reduced dopaminergic neuron death, decreased α-syn phosphorylation, reduced peripheral macrophages infiltration, improved motor function, and the prevention of the reduction of perineuronal nets. This suggests that in disease conditions, microglia can lose the ability to regulate ECM components, reflecting a toxic gain of function in PD [30]. Collectively, these findings point to an early interplay between α-syn and neuroinflammation, providing insights on the role of microglia in both the initiation and progression of PD. Therefore, many studies are focusing on understanding what drives microglia’s harmful response in PD.

However, the intricate response of microglia in Parkinson’s disease (PD) is anything but straightforward. It can be complex and driven by multiple factors, leading to a wide range of research approaches and varying findings. Hence, in the SN of a PD mouse model, microglia inflammatory responses were found to be stimulated by an α-syn-mediated release of the thrombolytic protease tissue plasminogen activator (tPA). This resulted in microglia activation, T-cell infiltration and dopaminergic neuron death [31]. In addition, ferroptosis, an iron-dependent form of programmed cell death, also had a share in microglia activation. In aged individuals, and particularly in PD patients, iron accumulates in brain areas that are vulnerable to neurodegeneration such as the SN. Increased free levels of Fe^2+^ in the cytoplasm of microglia modify its phenotypes to more pro-inflammatory ones, leading to an increased secretion of TNF-α and IL-6, and an increase in production of ROS in a NOX-dependent manner, resulting in α-syn overexpression and aggregation [32]. Adding to the complexity, the cell necroptosis executor protein MLKL (Mixed-lineage kinase domain-like) has been shown to be responsible for microglia neuroinflammatory and neurodegenerative effects in a progressive model of PD [33]. Meanwhile, environmental factors also weigh in—just as in AD, excessive copper exposure due to heavy metal pollution has been reported to be toxic for dopaminergic neurons. Cu(II) activates microglia via the NF-κB/p65 pathway, triggering neuroinflammation (NLRP3, IL-1β, and IL-18 release) and α-syn aggregation [34].

#### 2.1.3. Stroke

Stroke ranks as the third leading cause of death globally, with the majority of cases caused by an obstruction of blood flow in the brain and a consequent ischemic event [35]. Ischemic stroke leads to neuronal necrosis and to the activation of the innate immune response. This inflammatory response is a critical factor in both acute and chronic phases, but the nature and impact of inflammation differ between these two stages.

During the acute phase of ischemia, which occurs within hours of the event, the brain undergoes a strong inflammatory response. Activated microglia release pro-inflammatory cytokines, which then recruit peripheral immune cells like neutrophils, monocytes, and macrophages to the injury site. Neutrophils infiltrate the ischemic area rapidly, releasing ROS and proteolytic enzymes that worsen tissue damage and disrupt the BBB, leading to edema and further immune cell infiltration [36,37,38]. Although this response aims to clear dead tissue, it can extend the area of infarction and worsen the prognosis of the stroke [39]. Alternatively, chronic low-level inflammation, driven by glial cells, contributes to long-term neurological deficits and impaired recovery after stroke [40]. Balancing beneficial and detrimental effects of inflammation in this phase is complex, and therapeutic strategies aim to enhance reparative processes while mitigating the chronic inflammatory damage.

The outcome of inflammation in stroke is influenced not only by the acute/chronic phase of microglial activation but also by the distinct functional roles of specific microglial subsets. Current findings identified two unique microglial subsets in ischemic regions 3 days after stroke; CH25H^+^ and OASL^+^ microglia. CH25H^+^ microglia exhibit neuroprotective properties by promoting phagocytosis, neural network reconstruction, and neurogenesis, partly through the action of the enzyme CH25H, which modulates inflammation and mitochondrial integrity. Conversely, OASL^+^ microglia are pro-inflammatory, with an upregulated interferon response and a significant association with increased infarct volume, particularly in aged mice. This emphasizes the dynamic and specialized roles of microglia subclusters, and renders the study of stroke-related inflammation very challenging [41].

### 2.2. Border-Associated Macrophages (BAM)

BAMs are a recently characterized population of brain-resident immune cells distinct from microglia, and thus remain relatively underexplored in comparison. Despite this, they hold significant potential as a target for immunomodulatory strategies in the treatment of neurodegenerative diseases. The general roles of BAMs in physiological conditions are phagocytosis/glymphatic drainage, immune surveillance against infectious pathogens, and BBB maintenance [42]. These functions are highly related to their molecular heterogeneity and to their poorly documented interactions with their cellular environment. BAMs subtypes can be distinguished on the basis of LYVE-1 and MHCII expression [43]. Thus, MHCII^+^BAMs exhibit a pro-inflammatory profile due to their strong expression of MHCII and pro-inflammatory genes, such as *Cxcl9*, *Cxcl10*, *Cxcl13*, *Cxcl16*, *Irf5*, *Il1a/b*, *Cxcr4*, *Il2ra* and *Tlr2*, whereas LYVE-1^+^BAMs exhibit an anti-inflammatory profile due to their anti-inflammatory/immunosuppressive gene expression: *Ccr1*, *Cd163*, *Cd209a/f*, *Cd302*, *Igf1*, *Il21r*, *Mrc1*, *Stab1*, *Tgfb1*, and *Tslp* [44]. Signaling pathway analysis reveals that LYVE- 1^+^BAMs are enriched in genes associated with endocytosis and lysosomal activity, while MHCII^+^BAMs are enriched with genes related to antigen presentation and processing [43].

#### 2.2.1. Alzheimer’s Disease

Under pathological conditions such as AD, BAMs can play a dual role in disease progression. On one hand, they can contribute to neuroprotection by clearing Aβ deposits. On the other, they can aggravate pathology through inflammatory and oxidative mechanisms. BAMs are initially protective, but Aβ plaque deposition in both the parenchyma and vasculature disrupt their environment leading to molecular changes [44]. In response, BAMs modulate their molecular expression by downregulating the LYVE-1 receptor on their surface, thus losing their anti-inflammatory state [44]. Notably, similar molecular modulation has been demonstrated in aged mice [45], suggesting that age-related downregulation of LYVE-1 in BAMs may contribute to both their dysfunction and the onset of late-onset AD.

Conversely, BAMs can drive neurovascular damage, exacerbating AD-related complications. During CAA, BAMs confronted with Aβ40 are responsible for the increased production of CD5L^+^ migrasomes, which promote complement activation and BBB damage [46]. They also contribute to cerebrovascular dysfunction during amyloid immunotherapy by responding to immune complexes formed between anti-Aβ antibodies and CAA [47]. This activation of BAMs increases BBB permeability via the release of matrix remodeling factors like MMP9 and Timp1, which disrupt vascular integrity, facilitate monocyte infiltration into the brain parenchyma, and lead to microhemorrhages [47]. In addition, BAMs binding to Aβ via the CD36 receptor activate NOX2 (ROS -producing enzyme) and leads to ROS-mediated cerebrovascular dysfunction [48,49,50].

Given their detrimental role in neurovascular dysfunction, BAMs have been explored as therapeutic targets, though interventions have revealed both beneficial and detrimental effects. BAM depletion using clodronate liposomes (CLO) has been shown to attenuate Aβ-induced oxidative stress, BBB dysfunction, and cognitive decline, further highlighting their potential harmful role. However, BAM depletion is not without consequence [49,50], as it leads to excessive Aβ accumulation in the cortex [45] and exacerbates both tau pathology and tau-dependent neurodegeneration [51], underscoring their essential function in limiting disease progression.

Beyond their impact on the neurovascular unit, specific BAM subtypes, such as LYVE-1^+^PVM subtypes, have been implicated in mediating microglial synapse phagocytosis in the hippocampus of the *App*^NL-F^mouse model of AD. Aβ deposits on the cortical vessel wall are able to trigger phosphoprotein 1 (SPP1) production by PVMs, which in turn activate C1q in microglia and initiate synapse engulfment. Although this action can be associated with synaptic homeostasis and tissue remodeling or synaptic loss and dysfunction, its precise role—detrimental or beneficial—in AD remains to be fully determined [52]. Thus, BAMs exhibit a paradoxical role in AD, acting as both protectors and contributors to pathology depending on their state and interactions within the disease environment.

#### 2.2.2. Parkinson’s Disease

Interestingly, a critical role for BAMs has been pointed out in PD, with a focus on α-syn-mediated processes. BAMs, rather than microglia, were shown to mediate the recruitment and activation of CD4+T cells, facilitating immune infiltration into the CNS and initiating inflammatory cascades. The study identifies a disease-associated activation state in BAMs (DaBAMs) characterized by altered gene expression related to antigen presentation, T cell recruitment, and ECM remodeling. BAM depletion reduced markers of neuroinflammation and peripheral immune cell entry, highlighting their central role in PD pathogenesis. Postmortem analysis of PD brains revealed enhanced interactions between BAMs and T cells in perivascular spaces, implicating these processes in human disease [53]. scRNAseq data showed that α-syn-induced neuroinflammation is not totally dependent on microglia specifically for antigen presentation or for the infiltration of peripheral immune cells. They found that BAMs highly expressed several genes involved in antigen presentation (*H2-Aa*, *Cd74*, and *Cd274*), ECM remodeling (*Mmp14*), and T cell recruitment (*Ccl5* and *Ccl10*). Some expressed genes in BAM clusters involved in phagocytosis (*Cd68*), and others expressed genes involved in inflammation (*Il1b*) and lymphocyte chemotaxis (*Ccl5*, *Cxcl10*) [53]. These findings collectively suggest that BAMs, rather than microglia, play a pivotal role in α-syn-induced neurodegeneration. Consequently, targeting BAMs may represent a promising therapeutic strategy for modulating inflammation and neurodegeneration in PD.

#### 2.2.3. Stroke

In the acute stage of an ischemic stroke in rat brains, CD163^+^PVMs upregulate genes associated with the ECM remodeling, inflammation, and immune response, and downregulate the genes responsible for the inhibition of leukocyte activity. CD163^+^PVM presence is also shown to aggravate neurological dysfunction, secrete chemokines to induce leukocyte recruitment to the ischemic site, and increase the vasculature leakage in a vascular endothelial growth factor (VEGF)-dependent manner [54]. Interestingly, VEGF is detected around CD163^+^PVMs in human post-mortem brain patients who died 24 h after an ischemic stroke, validating the findings obtained in rats [54].

## 3. The Dual Role of Neuroinflammation in Shaping Neurogenesis

Inflammatory signals play a pivotal role in influencing the behavior of neural stem cells (NSCs), with chronic or excessive inflammation particularly impairing their proliferation, differentiation, and survival. This effect is notably significant in the context of neurodegenerative diseases, where persistent inflammation often disrupts normal regenerative processes. Glial cells and macrophages are central to this process, as they can either promote or hinder stem and progenitor cell function depending on their activation state. For example, pro-inflammatory cytokines such as TNF-α, IL-1β, IL-6, and IFN-γ negatively impact MSC viability and function. In contrast, anti-inflammatory cytokines such as IL-10, TGF-β, and VEGF-A promote MSC survival and differentiation. Additionally, cytokines like IL-6 and Oncostatin M, have been shown to stimulate osteoblast differentiation while inhibiting adipogenesis [55]. Conversely, IL-1β in bone injury models has been reported to inhibit MSC proliferation, migration, and osteoblastic differentiation, further complicating the regenerative process [55]. The influence of inflammation on neurogenesis is complex and context-dependent, as individual cytokines may exert opposing effects depending on the timing, source, and concentration (see Table 1). This underscores the critical role of these inflammatory signals in determining neuronal fate during neuroimmune insults, highlighting the need for careful modulation of the inflammatory environment in therapeutic strategies [55].

## 4. Modulating Neuroinflammation

Effectively modulating neuroinflammation to mitigate neurodegenerative diseases without exacerbating pathology remains a significant challenge for emerging therapies. Recent strategies show promise, including the administration of anti-inflammatory agents, bioactive compounds (Table 2), and either mesenchymal stem cells (MSCs) or MSC-derived exosomes with immunomodulatory properties. However, further research is required to optimize their therapeutic potential and clinical translation. Below, we explore the most recent and promising advancements in this field.

### 4.1. Anti-Inflammatory Bioactive Substances

#### 4.1.1. Glycolysis and Lactate Modulation

Understanding the causes of microglial inflammatory activation paves the way for developing treatments to overcome and modulate these harmful effects. Microglial activation undergoes a metabolic shift from oxidative phosphorylation to aerobic glycolysis in response to inflammatory triggers like LPS, α-syn, or Aβ plaques. Lactate, a metabolic by-product of this glycolysis reaction, has been observed to be elevated in the CSF of AD and PD patients [77,78], and to be involved in modulating gene expression linked to microglial activation and polarization [79]. Inhibiting the glycolysis pathway attenuates microglia-mediated neuroinflammation, resulting in the improved spatial learning and memory in AD mice [80] and in the protection of dopaminergic neurons in PD mice [79,81,82]. In acute ischemic stroke, similar to AD and PD, lactate levels significantly increase due to anaerobic glycolysis, shifting microglial metabolism to a pro-inflammatory state [83]. This rise in lactate is associated with a deficiency in the suppressor of MEK1 (SMEK1) in microglia, and modulating SMEK1 expression could provide a potential therapeutic target [83]. Lactate’s effects, however, vary depending on its concentration [84,85,86]. At low or moderate levels, lactate can have neuroprotective effects by reducing cerebral infarct volume and preventing neuronal apoptosis, partially through the activation of HIF-1α. This pathway suppresses the NF-κB pathway in microglia, promoting neuroprotection [86]. Moderate lactate levels help maintain microglial lysosomal function, clearing damaged cells and limiting inflammation. Therefore, therapeutic strategies may focus on optimizing lactate levels to harness its dose-dependent neuroprotective benefits.

#### 4.1.2. Chemical and Metabolic Compounds

NLRP3 inflammasome activation leads to caspase-1-dependent secretion of pro-inflammatory cytokines IL-1β and IL-18, and has been linked to AD. DAG-MAG-βHB, a promising neuroprotective ketone diester, has been shown to impair the NLRP3 inflammasome assembly in response to hypoglycemia and Aβ. DAG-MAG-βHB was also confirmed to be of a non-toxic nature, which is to preserve the BBB integrity in vitro as well as enhance microglial function and restore phagocytosis in order to clear debris [87]. In addition, treatment with chemerin-9 (an adipokine) significantly decreases NLPR3 inflammasome activation and increases phagocytic ability in microglia. This ameliorated APP/PS1 cognitive impairment attenuated neuronal and synaptic damage and relieved Aβ burden [88]. Other compounds, like PAP-1 (5-(4-Phenoxybutoxy)psoralen), a selective Kv1.3 potassium channel inhibitor, are showing promising neuroprotective effects. Kv1.3 channels are upregulated in activated microglia, particularly in response to α-synuclein aggregates (αSynAgg) in PD and other inflammatory stimuli. PAP-1 effectively reduces the release of pro-inflammatory cytokines, including IL-12, TNF-α, IL-1β, and IL-6, thereby mitigating neuroinflammatory damage [89].

#### 4.1.3. Pharmacological Drugs

AD-16 is an orally administered, safe, and tolerable anti-inflammatory drug used to treat AD patients [90], to modulate microglial neuroinflammation, and reduce infarct volume in mice models of neonatal [91] and adult cerebral ischemia [92]. In a PD-mouse model, AD-16 treatment given after the motor symptoms’ confirmation relieved motor impairments, reduced neurodegeneration of dopaminergic neurons, and alleviated the brain pro-inflammatory environment by reducing IL-1α and TNF-α in the SN pars compact and IL-1α, IL-1β, IL-6, and TNF-α in the striatum. Additionally, it lowered microglial density and restored their branched, star-like morphology (indicative of an inactivated state), confirming the attenuation of inflammation [93].

In ischemic stroke, microglial activation and NLPR3 inflammasome formation are linked to mitochondrial (mt) activities. Cytidine/uridine monophosphate kinase 2 (CMPK2), a key enzyme for mtDNA replication, is upregulated in the peripheral blood of stroke patients, and its expression correlates with infarct volume. In rodent models, CMPK2 expression increases in the peri-infarct region, promoting Ox-mtDNA-induced NLRP3 inflammasome activation, which exacerbates cerebral ischemic injury. Pharmacological inhibition of CMPK2 using nordihydroguaiaretic acid (NDGA), a natural anti-oxidant, suppressed the hyperactivated state of microglia, limited mtDNA oxidation, reduced NLPR3 inflammasome production, and mitigated brain injury [94]. Additionally, cathepsin S (CTSS), a lysosomal protease predominantly expressed by microglia and upregulated after stroke, contributes to BBB leakage and neuroinflammation [95,96,97]. Inhibition of CTSS with benzydamine, a nonsteroidal anti-inflammatory drug (NSAID), blocks the p65/NF-κB pathway, reduces neuroinflammation, and improves ischemic injury outcomes [98].

The stimulator of interferon genes (STING) act with cyclic GMP-AMP synthase (cGAS) to shift microglia to a pro-inflammatory state by inducing autophagy and activating the type I interferon signaling pathway [99,100]. In cerebral ischemia, this cGAS-STING signaling pathway can be activated by ferroptosis [101] and can be inhibited using STING-inhibitor H-151. This STING inhibition alleviated the production of pro-inflammatory cytokines (TNF-α and IFN-γ), decreased infarct volume [100], and resulted in ameliorated motor coordination and cognitive learning defects [99].

#### 4.1.4. Cytokines and Peptides

IL-10 gene therapy, delivered specifically to microglia in the SN pars compacta, induced the expression of genes associated with key factors of phagocytosis and α-syn clearance contributing to neuroprotective benefits. This IL-10-driven microglial activation also reprogrammed T cells into regulatory T cells (Tregs), which help suppress harmful immune responses in the brain [102].

On the other hand, mesencephalic astrocyte-derived neurotrophic factor (MANF) activated the autophagic system, resulting in the inhibition of SN α-syn accumulation and LPS-induced neuroinflammation [103]. Another promising treatment includes recombinant human fibroblast growth factor 21 (rhFGF21). RhFGF21 reduces inflammation in microglia and macrophages via NF-κB and PPAR-γ signaling, decreasing infarct size and peripheral immune cell infiltration [104,105].

#### 4.1.5. Plant Based Compounds

Plant-derived anti-inflammatory compounds are widely being used due to their safety and low side effects. Cedrol, a natural sesquiterpene alcohol from ginger, has been reported to suppress the pro-inflammatory response in LPS-stimulated microglia in an acute stroke mouse model. It reduces infarct size and improves behavioral outcomes by interacting with estrogen receptor β (ERβ), blocking the p65/NF-κB signaling pathway and suppressing inflammatory cytokine production [106,107]. Similarly, Ligustilide, an active compound from traditional Chinese medicine, effectively treats neuroinflammation by penetrating the BBB, downregulating the FPR1/NLRP3 signaling pathway, and suppressing inflammatory mediators, providing neuroprotective effects [108].

Other compounds such as Frictus Tribuli (FT) and kaempferol (KAE), which are traditional medicines that have been used for thousands of years, were found to reduce neuroinflammation in in vivo PD models [109,110,111]. FT inhibited microglia and astrocyte activation, protected dopaminergic neurons, increased dopamine levels, and reduced bradykinesia. It also regulated the c-Jun N-terminal kinase (JNK) pathway, which is involved in synaptic dysfunction, neuronal apoptosis, and memory deficits [112]. KAE administration significantly reduced the activation of microglia and astrocytes, as well as the levels of iNOS and COX-2. KAE was suggested to exert anti-pyroptotic effects over microglia by inhibiting the NLRP3 inflammasome through downregulation of the p38MAPK/NF-κB signaling pathway [111]. It is worth noting that KAE also showed promising effects for the treatment of AD and cerebral ischemia [110,113].

Amyloidosis in AD is influenced by lipopolysaccharides (LPS), which upregulate iNOS, contributing to disease progression. LPS binding on the toll-like receptor 4 (TLR4) was highly expressed on microglia, and it induced the latter to release ROS and pro-inflammatory cytokines such as IL-1β and TNF-α. Interestingly, the use of a novel highly bioactive vanadium-curcumin complex has been shown to disrupt LPS-induced amyloid precursor protein (APP) and alleviate iNOS and pro-inflammatory cytokines production, rendering curcumin-based compounds a promising therapeutic approach [114].

#### 4.1.6. Ultrasound

Ultrasound, a diagnosis tool, is nowadays being used to treat diseases [115,116] due to its ability to penetrate deeply in the tissue and to precisely focus on injured sites to achieve the targeted activation and polarization of microglial cells [117]. In pre-ischemic mice brains, pretreatment with low-intensity pulsed ultrasound (LIPUS) 15 min daily for 5 days significantly improved apoptotic cell death and brain damage via BDNF production [116]. Low-intensity transcranial focused ultrasound (tFUS) also promotes neurorehabilitation by suppressing NLRP3 inflammasome and increasing microglial anti-inflammatory cytokines production [118,119]. Additionally, a hybrid microglia-based therapeutic platform combining ultrasound-responsive IL-4-loaded liposomes with engineered microglia has demonstrated promising results. By fusing platelet membranes with microglia (PM-MG), the hybrid cells gained the natural injury-targeting ability of platelets to migrate to injured cerebral vessels. Upon ultrasound stimulation, a controlled release of IL-4 at the injury site enhanced BDNF expression, promoted neuron regeneration, suppressed inflammation, and repaired the BBB in stroke [117].

### 4.2. Priming, Training the Immunity

Regenerative medicine research has shown that the therapeutic benefits of MSCs stem from their secretion of bioactive factors rather than direct cell replacement. New approaches focus on genetically modifying stem cells using methods like epigenetic priming, enabling them to generate specific anti-inflammatory mediators and neurotrophic factors. Preconditioning these cells maximizes their clinical potential by improving their therapeutic effectiveness in challenging inflammatory environments.

Due to the opposite effect of pro- and anti-inflammatory immune cells after brain damage [120], cellular priming (also known as cell preconditioning or licensing) is an emerging strategy for brain repair. It consists of dictating the cell-specific differentiation, activation, and phenotype changes; function; and molecular signalling. Immune cells and progenitors in culture can be trained by adding specific cytokines to their medium to be polarized into a pro- or anti-inflammatory nature [121]. For example, monocyte-derived macrophages primed into anti-inflammatory phenotype and transplanted into the CSF following focal ischemia led to post-stroke recovery of motor and cognitive function [122].

Pretreating MSCs with pro-inflammatory factors enhances their paracrine activity and strengthens their immunosuppressive properties [123]. This enables them to produce anti-inflammatory and neurotrophic factors [121]. The use of conditioned media (CM)—the secretome of MSCs containing cytokines, growth factors, and various bioactive molecules—has emerged as a cell-free therapeutic approach for a wide range of diseases [123]. For example, in a PD mouse model, the secretome of menstrual-cycle-derived stem cells (MenSC), was shown to reduce cytotoxicity, inflammation, oxidative stress, and mitochondrial damage [124]. This effect was due to its composition of more than 12 neurotrophic factors [124,125]. Given that the therapeutic effect of MSC is attributed to secreted bioactive factors rather than to cell replacement, its secretome potential is particularly promising in the treatment of brain inflammation in neurodegenerative disorders [123,126].

In AD research, MSCs and their secreted factors have shown promise for therapeutic use. In an in vivo rat model of AD, CM from hypoxic preconditioned MSCs have been shown to decrease brain inflammation (reduction of IL-1β and TNF-α) and Aβ plaques, and to improve neuronal survival and memory deficit [127]. Similarly, in an in vitro model of AD, secretome from unconditioned MSCs showed neuroprotective, anti-inflammatory, and antiapoptotic properties by activating the Nrf2/ARE antioxidant pathway and supporting neuronal differentiation via neurotrophic factors. Further studies revealed that preconditioning MSCs with CoCl_2_ (hypoxia mimetic) or TLR3 activation enhanced CM’s efficacy by increasing the expression of immunosuppressive/immunomodulatory (IDO1, TNFAIP6, and PTGES2) and neurotrophic molecules (VEGF, IL-4, IL-10, and TGF-β) [123]. Notably, these preconditioned CMs also reduced oxidative stress, and shifted the immune cell dynamic into an anti-inflammatory phenotype. Together, these findings underscore the potential therapeutic effects of MSC-derived CM in targeting neuroinflammation, cellular survival, and oxidative stress in neurodegenerative diseases, especially AD [127,128,129,130].

MSC priming with α-syn have exhibited an augmented stemness and an enhanced secretion of exosomes packed with autophagy-regulating miRNAs (miR-376b, miR-374b, and miR-7-5p). This α-syn-mediated MSC modulation induced autophagy and lysosomal activity that aided the clearance of α-syn enriched neurons. Similarly, animal studies showed that α-syn-primed MSC generated higher numbers of dopaminergic neurons in the SN, supporting the potential of α-syn priming to augment MSC stemness and neuroprotective effects, offering a promising therapeutic strategy for PD [131]. In another approach, uric acid (UA)-primed MSCs were tested in both cellular and Parkinsonian mouse models. The treatment effectively reduced apoptosis in dopaminergic neurons, as evidenced by decreased cleaved caspase-3 expression. Additionally, UA-primed MSCs modulated inflammatory responses, increasing anti-inflammatory cytokines and reducing pro-inflammatory cytokines. Notably, these MSCs also lowered the expression of miR-137 and miR-145, potentially enhancing MSC stemness through the activation of key transcription factors such as OCT4, NANOG, SOX2, and KLF4 [132].

FGF21 has been shown to suppress neuroinflammation and protect dopaminergic neurons. However, due to its short half-life in the brain, repeated injections are required for sustained effects. To address this, researchers have genetically engineered MSCs to overexpress FGF21, enabling safer intranasal delivery. In PD mouse models, intranasal administration of FGF21-overexpressing MSCs before or after PD induction effectively alleviated motor symptoms, reduced dopaminergic neuronal death, and restored brain-derived neurotrophic factor (BDNF) levels in the SN. In PD cell models, conditioned medium from FGF21-overexpressing MSCs or recombinant FGF21 reversed dopaminergic neuron death, reduced mitochondrial ROS, and enhanced the levels of phospho-Akt, mature BDNF, and Bcl-2, underscoring the therapeutic potential of FGF21-based MSC treatments for PD [133].

Similar to AD and PD, stroke has also been a central focus for CM therapies [134,135,136]. In focal cerebral ischemia, pre-treatment with a CM derived from human amniotic membrane (AMSC-CM) has reduced lesion volume and BBB breakdown, and improved motor and neurological outcomes across the acute, subacute, and chronic phases [137]. Preconditioning MSCs with IL-1α induced an anti-inflammatory, pro-trophic phenotype that enhanced their regenerative potential, with IL-1α-derived MSC-CM improving stroke outcomes when administered alongside thrombectomy [138,139,140]. In mild stroke models, single or repeated administration of human dental pulp stem cell (hDPSC) secretome showed short-term benefits [141]. Additionally, in an aneurysmal subarachnoid hemorrhage model, DPSC derived secretome limited brain edema formation, improved microcirculation, enhanced functional recovery, and polarized microglia to an anti-inflammatory phenotype partially through IGF-1/AKT signaling [142].

The secretome from neural precursor cells (NPC), administered in a permanent MCAO rat model, significantly reduced infarct size, improved behavioral outcomes, and modulated microglial polarization. It also mobilized endogenous neural progenitors and induced neurogenesis while upregulating anti-inflammatory cytokines (IL-4, IL-10, BDNF) and genes related to neurogenesis, CNS development, and synaptic transmission. Simultaneously, it downregulates pathways involved in inflammatory response, ECM organization, macrophage activation, and collagen fibril organization. Notably, multiple NPC secretome injections amplified these effects considerably [143].

### 4.3. Exosomes

Exosomes are small vesicles (30–150 nm) that facilitate intercellular communication by mediating crosstalk between multiple cells and shuttling cargo between the cells and tissues. They allow more precise therapeutic interventions by specifically binding to target cells [144]. These exosomes contain bioactive molecules such as nucleic acids (long-chain non-coding RNAs (lncRNAs), microRNAs (miRs)), proteins, lipids, and metabolites [144,145]. Since exosomes originate from cells, they have low immunogenicity and toxicity, meaning they do not provoke immune responses or cause harmful side effects when utilized as a therapeutic option [144,146]. Therefore, they mirror the characteristics of their cells of origin, offering therapeutic potential comparable to their donor cells for cell-free treatments [144,145]. Compared to traditional gene therapy vectors, exosomes offer significant advantages, such as superior targeting, RNA transport capacity, stable presence in body fluids, capability of crossing the BBB, safety, low risk of tumor formation, and finally diverse administration methods, such as inhalation [147].

In the context of neurological disorders, these membrane-bound vesicles are actively released from NSCs and microglia, particularly following brain injury [148]. Exosomes from these cells carry specific proteins and miRNAs that influence inflammation, neurogenesis, and tissue repair. Microglial exosomes enriched with markers like CD13 and inflammatory miRNAs such as miR-155 can modulate NSC proliferation, differentiation, and inflammatory responses, sometimes contributing to neurotoxicity. Conversely, NSC exosomes, characterized by markers like CD63 and miR-126, suppress inflammation, enhance neuroprotection, and promote recovery. Despite their dual roles in injury and repair, exosomes show immense therapeutic potential, warranting further research to elucidate their mechanisms and explore their application in treating brain injuries [148].

Beyond brain injury, in neurodegenerative diseases, exosomes contribute to disease progression by aiding in the removal of infectious and cytotoxic materials from cells and by facilitating the intercellular transfer of pathogenic materials. In AD, exosomes transport full-length Aβ precursor protein (flAPP), APP metabolites, and cleavage enzymes into the extracellular space accumulating in Aβ plaques and aggravating the disease [149,150]. Similarly, in PD, exosomes propagate pathology by transporting mutated and toxic oligomeric forms of α-syn, leading to motor deficits [151].

Despite their role in neurodegeneration, exosomes also hold great promise for therapeutic applications. Exosomes derived from MSCs can have advantageous effects in various contexts, including neurological, respiratory, cartilage, kidney, cardiac, and liver diseases, bone repair, and cancer. In AD, MSC-exo can reduce neuronal cell apoptosis, promote nerve regeneration, and protect against glutamate excitotoxicity. It can serve as a smart drug delivery approach through the transportation of exogenous chemicals and biomolecules for stem cell-free regenerative medicine. For example, exosomes can assist in degrading Aβ by containing enzymes like Neprilysin (NEP) and cystatin C. They can also stimulate neurogenesis in the subventricular zone and alleviate Aβ 1-42-induced cognitive impairment [152,153]. Similarly, NSC-derived exosomes can significantly reduce Aβ levels by promoting non-amyloidogenic processing of APP through increased ADAM10 (α-secretase) activity and by inhibiting BACE1 (β-secretase) and PSEN1 (γ-secretase) activities [154]. Their ability to carry RNA molecules, such as miRNA and siRNA, allows them to be used as delivery vehicles for gene therapy [147,153,155]. Intranasal administration of EVs has emerged as an efficient means to enhance brain delivery, allowing exosomes to enter easily and efficiently the brain, and it can also be taken up by neurons to exert neuronal proliferation, lower Aβ deposition, and improve spatial learning and memory function [156].

In PD, engineered EVs have demonstrated promising results. Using a crosslinking reaction, dopamine was conjugated to the surface of EVs to specifically target dopaminergic neurons in PD brains. Dopa-EVs, injected intravenously, accumulated at a high concentration in the brain, mitigated neurodegeneration, decreased α-syn accumulation, and improved cerebral ataxia. Dopamine only served to target the neurons, but the effect observed was due to the EVs content [157]. Similarly, human umbilical cord (UCB) MSC-derived exosomes were loaded with BDNF and were delivered intravenously in the PD mouse model. Once administered, BDNF-exosomes successfully crossed the BBB, reached affected areas of the brain, and enhanced neuronal survival. BDNF-exosomes also promoted neuronal cytoskeletal stability and enhanced antioxidant defense, ensuring neuroprotection against damage [158]. In addition to this, UCB-Exos significantly inhibited the MAPK p38 and ERK 1/2 pathway and down-regulated the expression of P21, P27, and P53 genes, thereby weakening dopaminergic neuron injury and ameliorating the symptoms of PD [159]. Subsequently, UCB-exos suppressed inflammatory factors and increased gene expressions related to NSC proliferation and differentiation [159].

### 4.4. Biomaterials for Controlled Inflammation

The delivery of MSCs and MSC-derived exosomes or secretomes can be facilitated using biomaterials and scaffolds such as hydrogels or collagen. While collagen is a key component of the ECM, it degrades rapidly. To address this, Collagen can be coupled to a scaffold carrier ‘’Chitosan’’ that delays collagen degradability and enhances its mechanical strength. In a traumatic brain injury (TBI) study, a 3D-printed and optimized collagen-chitosan (CC) scaffold was designed to maximize exosome loading while preserving their viability and bioactivity. The scaffold enabled uniform exosome distribution and sustained a higher release rate. Subsequently, exosomes from IFN-γ-conditioned NSCs (IFN-γ-exo) were incorporated into this CC scaffold and administered in the injured brain area. This CC- IFN-γ-exo scaffold enhanced NSC differentiation and endogenous neurogenesis while also facilitating vascular remodeling. Additionally, it alleviated neuroinflammation by modulating microglial activation and promoting the transition to an anti-inflammatory phenotype via the MAPK/mTOR signaling pathway [160].

Hydrogels are being widely used due to its low mechanical stiffness, its availability in various sizes (bulk gels, microgels, and nanogels), its tissue-like three-dimensional (3D) environment, and its non-invasive administration [35,161]. Hydrogel scaffolds can control and ensure the sustained release of MSC-exosomes and anti-inflammatory molecules. For example, a gelatin methacryloyl (GelMA) hydrogel was used to 3D culture MSC to prepare 3D-MSC-Exo, which exhibited enhanced neuroprotective effects, including reduced neuroinflammation, inhibition of glial scarring, and promotion of angiogenesis and neovascularization [162,163]. In addition, MSC encapsulated in gelatin hydrogel showed promising effects for the treatment of chronic cerebral ischemia by promoting neovascularization, facilitating neuronal differentiation, inhibiting early apoptosis, and suppressing neuroinflammation [164]. In MCAO mice, a combined therapy of a hyaluronic acid hydrogel scaffold modified by catechol and loaded with exosomes showed a decrease in inflammation and improvement in the neurovascular remodeling, angiogenesis, and infarct volume [161].

## 5. Stem Cell-Derived Models for Neuroinflammation

**Figure 2:**
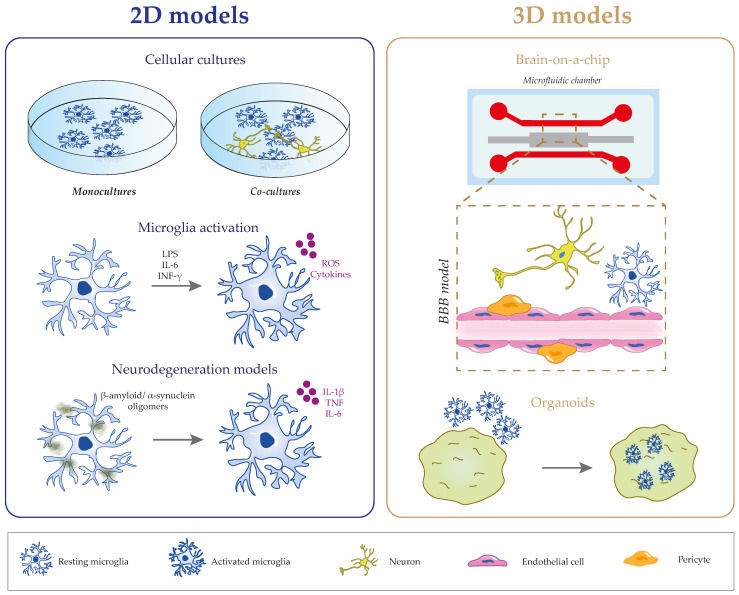
Scheme summarizing stem-cell-derived in vitro models used for the study of neuroinflammation. Two-dimensional models (**left**) include monocultures and co-cultures, where microglia activation is induced by stimuli such as lipopolysaccharide (LPS) or disease-related factors like β-amyloid. In contrast, 3D models (**right**) include brain-on-a-chip systems that allow the modeling of the blood-brain barrier (BBB) and brain organoids.

Modelling neuroinflammation using stem-cell-based in vitro approaches has emerged as a powerful tool for understanding the complex interactions between neural and immune cells in brain disorders, particularly neurodegenerative diseases. Despite animal models having been widely employed, they often fail to fully recapitulate human-specific inflammatory responses. Key aspects of immune responses, BBB biology, and inflammatory processes differ between humans and animals, limiting the translatability of findings and hindering the development of effective therapies. Additionally, the high costs and ethical concerns of working with animal models further limit their utility in studying human neuroinflammation. This has led to a growing need for alternative approaches that better reflect human physiology. Human-induced pluripotent stem cells (iPSCs) and neural organoids provide a dynamic platform to study neuroinflammatory mechanisms in a patient-specific and physiologically relevant context. These models facilitate high-throughput screening, personalized medicine approaches, and the exploration of disease mechanisms in a controlled environment, ultimately boosting our understanding of neuroinflammatory processes and the development of targeted therapies. Over recent years, several in vitro models have been developed to investigate neuroinflammation and brain immunity. These include monocultures of stem-cell-derived cells, co-culture systems, and three-dimensional (3D) models (scheme in Figure 2). Each approach enables the study of distinct experimental questions, offering specific advantages and limitations, which will be discussed in the following section.

### 5.1. Two-Dimensional Cultures

Two-dimensional (2D) monocultures are frequently used to investigate the role of specific cellular types in neuroinflammation. They allow for an in-depth analysis of cell morphology and the specific contribution of individual cell types to inflammatory responses under controlled conditions. Among these, microglial monocultures are the most commonly explored models. Primary murine microglial cultures have been instrumental in studying intracellular signalling pathways, cell interactions, and microglial activation during inflammation. They have provided valuable insights into cytokine release and responses to neurotoxic stimuli [165]. However, significant transcriptomic differences exist between human and murine microglia [166,167,168]. These differences limit the ability of mouse-derived microglial cultures to fully replicate human-specific functions in both physiological and disease contexts [169]. To address this challenge, alternative models, such as iPSC-derived microglia (iMG) have been proposed.

Different protocols have been optimized to generate microglia-like cells from iPSCs closely resembling human microglia functional properties and gene expression profiles [170,171,172,173,174,175,176,177,178,179,180]. These protocols mimic in vivo microglial differentiation by exposing hematopoietic precursors to key developmental cues via small molecules or cell reprogramming through inducible transcription factors [165]. The differentiated cells were validated by transcriptomic and proteomic analysis, showing high similarities with their in vivo counterparts. Indeed, it has been described that iMG exposed to brain-related substrates recapitulate the different transcriptional states seen in vivo including the appearance DAM-like states [169] dependent on TREM2 signalling or the regulation of disease-associated genes through MITF activity. Once differentiated and upon stimulation with various inflammatory triggers, such as LPS, interleukin-beta (Il-β), or interferon-gamma (INF-γ), these cells exhibit a robust immune response characterized by cytokine and ROS release, canonical inflammasome activation, and IFN-γ production [178,181]. The cells are also able to phagocyte different substrates ranging from *E. coli* particles or to more neuronal-relevant substrates such as synaptosomes [171,172,180,181]. In this direction, neurodegenerative disorders like AD or PD have been modelled through the incubation of the iMG with neurodegeneration-associated proteins. The stimulation of microglia cells with β-amyloid oligomers or α-synuclein oligomers induces an inflammatory phenotype in the microglia cells and increases the levels of IL-1β, TNF, and IL-6 [182,183,184].

While monocultures are useful for assessing the role of specific cell types in inflammation, they fail to fully capture the complexity of cellular interactions and the brain microenvironment. For example, microglia interactions with both neuronal and non-neuronal cells are crucial for proper differentiation and maturation. Moreover, the contacts established between neurons and microglia could modulate the active state of last ones [185,186,187]. This underscores the need for more sophisticated models that better reflect these dynamic cellular relationships.

To explore the effects of molecular interactions and signalling cues but not cell–cell contacts, conditioned media from a specific cellular type could be added to monocultures of another cell population, or special devices could be used to maintain the different population separated through porous membranes that allow molecule diffusion [188]. One example is the incubation of iPSCs cultures with neuronal or neuronal precursors conditioned media to improve the differentiation of iPSCs to microglia cells [188]. Alternatively, co-cultures could be generated, allowing direct cell–cell interaction between cell populations by plating more than one cellular type. It has been shown that the co-culture of microglia cells with other cellular types helps the maturation of the cells exhibiting phenotypes that more closely resemble in vivo conditions. Those models could also be applied to the study of neurodegenerative diseases. iPSCs-derived triple cultures of neurons, astrocytes, and microglia have been used to explore AD pathology [177,189], revealing that microglia could decrease neuronal death and increase Aβ plaque formation after being exposed to Aβ 1-42 oligomers [189].

### 5.2. Three-Dimensional Models

Although co-culturing systems offer many advantages compared to monocultures, there is still a lack of tissue architecture and neuronal heterogeneity that impedes a proper representation of the brain microenvironment, and 3D models have emerged as a promising tool to better recapitulate the in vivo landscape. Brain-on-a-chip (BoC) systems serve as platforms for studying physiological brain function and neurological disorders. These microfluidic-based models integrate diverse neural populations with perfusable vascular networks, offering more physiological relevance compared to traditional 2D in vitro cultures [190]. By optimizing nutrient and oxygen delivery, BoC models better replicate the brain’s microenvironment, while ECM components enhance signal transduction and cell–cell interactions. This improved biomimicry is reflected in transcriptomic profiles that closely resemble those of the human adult cortex, making BoC systems invaluable for studying neurobiology and disease mechanisms. Different studies have attempted to model neuroinflammation and neurodegenerative disorders using this microfluidics technology. Pediaditakis and colleagues developed a brain-on-a-chip model incorporating endothelial-like cells, pericytes, glia, and cortical neurons to replicate BBB permeability [191]. This advanced system exhibits gene expression profiles closely resembling the human cortex, outperforming simpler models in capturing key neurobiological pathways. Upon exposure to TNF-α, researchers observed glial activation, increased release of proinflammatory cytokines, and a significant compromise in barrier integrity, demonstrating its potential for studying neuroinflammation and BBB dysfunction [191]. Other examples of brain-on-a-chip models of neuroinflammation include the work of Schwartz and colleagues, who used synthetic hydrogels to self-assemble neural progenitors, endothelial cells, mesenchymal stem cells, and microglia/macrophage precursors, resulting in an interconnected vasculature with ramified microglia [192]. Berjaoui and colleagues designed a neurovascular unit-on-a-chip system to replicate the microenvironment of the BBB, where microfluidic systems allowed different cell types to interact across porous membranes [193]. Furthermore, human organotypic microphysiological systems incorporating endothelial-like cells, pericytes, glia, and cortical neurons have successfully maintained BBB permeability at in vivo-relevant levels while recapitulating key aspects of neuroinflammation [191]. BoC has also been explored to replicate neurodegenerative diseases, such as AD or PD in vitro, and to test new drugs [194]. Therefore, these organ-on-a-chip platforms represent a significant advancement in studying neuroinflammatory processes. However, they still lack the ability to self-organize into 3D structures, making it difficult to accurately simulate the complex brain environment and reproduce the neurodevelopment of the tissue.

Brain organoids offer several advantages over traditional 2D culture systems and brain-on-a-chip strategies, including increased cellular heterogeneity, more complex tissue architecture, and a microenvironment that better mimics in vivo conditions [195]. Unlike 2D cultures, which often lack the intricate organization of the brain, organoids enable the formation of distinct cellular layers and regions, supporting the development of neuronal circuits and interactions between multiple cell types. Additionally, they more accurately reproduce key cellular and molecular interactions, including neuron–glia communication, synapse formation, and extracellular matrix dynamics. This enhanced physiological relevance makes organoids valuable for studying neurodevelopmental processes, disease modelling, and drug screening, bridging the gap between in vitro cultures and in vivo studies. Organoids are derived from iPSCs using either unguided differentiation protocols, which result in the formation of the whole brain [196,197], or guided protocols that enable the generation of specific brain regions, such as the cortex or striatum [198,199,200,201,202]. The resulting structures contain stem cells, neurons, and glial cells. Although some unguided protocols may generate non-ectodermal cells due to the low restriction of cell identity [195], most protocols produce organoids that lack key cell types involved in the neuroinflammatory processes, such as microglia and endothelial cells. Recently, several protocols to incorporate microglia or microglia precursors into brain organoids have been published [203]. The microglia-brain organoids could be produced using different methods that varies in the microglial origins, stages, or proportions [203]. Moreover, microglial cells or progenitors could be co-cultured with brain organoids or with NPCs to form the final fused MC-BOs [203,204]. Additionally, the absence of a functional vasculature, which plays a crucial role in neuroinflammation and immune responses, significantly limits the potential of these 3D models. For this reason, different groups are exploring the introduction of a vascular network into the organoids through different approaches. Cakir and colleagues engineered hESCs to ectopically express the gene ETV2 resulting in the obtaining of cortical organoids with a vascular-like network [205] that presents blood-brain barrier characteristics. Sun and colleagues developed blood vessel organoids and fused them with brain organoids to obtain vascularized brain organoids [206].

Although it is a relatively new field that we are only beginning to explore in depth, there are already some studies in which organoids have been used as a model for neurodevelopmental and neurodegenerative disorders, including AD or PD. One of the first organoid models for Alzheimer’s disease was generated from mutated progenitor cells containing familial AD (fAD) mutations in the APP and PSEN1 genes, resulting in organoids presenting amyloid aggregation and hyperphosphorylated tau [207]. Subsequently, different groups have developed organoids from AD patients containing mutations in the APP, PSEN1, or APOE1 genes that recapitulate the main hallmarks of AD and have proven to be useful for drug testing [208,209,210,211,212,213]. In the case of Parkinson’s disease, midbrain-like organoids (hMLOs) have been derived from iPSCs with mutations in the principal genes identified as risk factors for this disorder (e.g: SNCA, LRRK2, PINK1, DNAJC6, PARKIN, ATP13A2, DJ-1). The organoids generated show α-synuclein aggregation, dopaminergic neuron-impaired differentiation or loss, and elevated oxidative stress levels, among others [214,215,216]. Apart from neurodegenerative diseases, brain organoids have also been used to model neurodevelopmental conditions or viral infections. However, modelling stroke presents significant challenges. Traditional 2D culture systems under hypoxic conditions have inherent limitations, primarily due to the absence of a functional vasculature, which is essential for accurately mimicking the ischemic brain environment. Only recently have 3D models emerged, offering a more physiologically relevant approach by better replicating oxygen gradients of the brain. Kim and colleagues developed neural organoids and exposed them to a hypoxic injury that led to alterations in the cellular composition and neuronal maturation [217]. In parallel, Wang and colleagues exposed cortical organoids to oxygen-glucose deprivation to generate an ischemic model that could be employed for drug testing [218]. Despite these advances, current organoid-based stroke models remain incomplete due to the absence of a functional vascular system. The incorporation of endothelial cells or vasculature to obtain more complex organoids would be a great improvement of those 3D models of stroke. The integration of endothelial cells or the development of vascularized brain organoids previously mentioned would represent a major breakthrough, allowing for more accurate modelling of stroke-induced ischemia, BBB dysfunction, and neurovascular interactions.

## 6. Conclusions and Future Perspectives

This review compiles the latest advancements in understanding neuroinflammation, with a particular focus on microglia and BAMs and their role in orchestrating immune responses in AD, PD, and ischemic stroke. While microglia have long been recognized as key regulators of the brain’s innate immune response, emerging evidence highlights the significance of BAMs’ contribution to neuroinflammatory processes. A comprehensive understanding of macrophage dynamics and their interactions with stem cells will yield critical insights into the mechanisms underlying neurodegenerative diseases, stroke, and other brain injuries.

Human stem-cell-based models offer a powerful platform to accelerate research in this field, enhancing translational potential and enabling more personalized therapeutic strategies. Future perspectives for 2D and 3D human iPSC-derived cultures in modeling neuroinflammation include advancing bioengineering techniques to enhance cellular complexity and microenvironmental cues, improving scalability for high-throughput drug screening, and integrating multi-omics approaches for deeper mechanistic insights. However, challenges remain, such as replicating the full cellular diversity of the brain, maintaining long-term culture stability, and achieving functional maturation comparable to in vivo systems. These models hold significant potential for complementing animal studies by providing human-specific insights into neuroinflammatory pathways, refining preclinical drug testing, and reducing reliance on animal models while improving translational relevance.

Therefore, advancing investigations in immune modulation will be essential for developing precise, targeted interventions to support brain health and promote regenerative medicine.

## Figures and Tables

**Table 1:** List of cytokines and their impact on neuroinflammation and stem cells neurogenesis.

Cytokines	Disease	Impact
IL-1β	Elevated in AD and MS Osteoarthritis Rheumatoid arthritis	Increases oxidative stress [56] Promotes a pro-inflammatory state by favoring pro-inflammatory astrogenesis [57] **Acute** IL-1β exposure contributes to hematopoietic stem cells (HSC) regeneration/**chronic** exposure promotes uncontrolled HSC division and exhaustion of the HSC pool [58] Induces MSC migration, adhesion and leukocytes chemotaxis migration via the NF-κβ [59] (human derived MSC) and independently from the NF-κβ pathway (murine derived MSC) [60] Enhances adipose and umbilical-cord-derived MSC ability to modulate macrophages toward an anti-inflammatory phenotype [61,62] Induces neuronal differentiation of neuronal precursor cells (NPCs) via the Wnt5a/RhoA/ROCK/JNK pathway [63]
Microglia Macrophages Astrocytes		
CXCL12	MS	Facilitates neurogenesis and tissue repair. Modulates NSC survival, proliferation, and migration [64] **Chronic MS lesion**: Promotes remyelination via CXCR4 activation on OPCs [64] **Active MS lesions**: CXCL12 depolarization at the BBB enhances leukocyte adherence to vessels and promotes leukocyte infiltration, thus promoting inflammation and demyelination [65]
Astrocytes Endothelial cells		
Tumor Necrosis Factor alpha (TNF-α)	AD, Stroke, PD, ALS and MS	**High concentrations** of TNF-α can inhibit the proliferation of NSCs and impair their differentiation into neurons. Prolonged exposure to elevated levels of TNF-α is associated with increased apoptosis (programmed cell death) in NSCs [66,67] May play a dual role in the NSC quiescent and active state [68] During NO-induced neurotoxicity, early endogeneous TNF-α was found to be neuroprotective [66] Triggers the immunosuppressive function of MSCs in inhibiting T cell proliferation [69]
Microglia (major source), neurons and astrocytes		
IFN-γ		Pro-inflammatory cytokine that drives NSCs away from a neurogenic fate and promotes glial differentiation, specifically astrocytosis [70] **Chronic** IFN-γ exposure leads to neurogenesis inhibition and impairs brain plasticity [71,72]
Cytotoxic T Lymphocytes (CTLs): CD8+ T cells		
IL-6	Traumatic brain injury (TBI), Stroke, AD	In TBI: In contrast to its potentially beneficial effects at acute or low concentrations, **chronic or elevated levels** of IL-6 can result in neuronal death, impair NSC proliferation, and favor astrogliogenesis over neurogenesis [73] In stroke: IL-6 plays a complex role in ischemic stroke, promoting post-stroke angiogenesis, neurogenesis, and long-term recovery, while reducing excitotoxicity, inflammation, and neuronal death. However, its elevated levels are also associated with stroke severity [74] Dysregulated IL-6 weakens the BBB, aggravating neuroinflammation, resulting in peripheral immune cells entering the brain and causing neuronal damage [74]
Microglia, Astrocytes, epithelial cells and neurons		
Reactive Oxygen Species (ROS) and Oxidative Stress		ROS levels play a dynamic role in determining the balance between NSC self-renewal, differentiation, and quiescence: High levels of ROS can induce NSPC death and quiescence. On the other hand, elevated ROS is associated with increased production of their immediate progenitors, NSC self-renewal and neurogenesis [75], and hESCs differentiation [76]
Mitochondria, peroxisomes, endoplasmic reticulum, and lysosomes as well as enzymatic reactions like those of NADPH oxidases (NOXs) and cyclooxygenase		

**Table 2:** Summary of bioactive substances used for modulating neuroinflammation: experimental models and dosing regimens in in vivo and in vitro studies.

Bioactive Substances	In Vivo or In Vitro
**Chemical and metabolic compounds**	
DAG-MAG-βHB	**in vitro:** HMC3 human microglia cell line primed with Aβ and low-glucose conditions (dose: 10mM)
Chemerin-9	**in vivo:** APP/PS1 mice (dose: 30 and 60 μg/kg body weight, daily for four weeks) **in vitro:** primary microglial cells collected from pups on post-natal day 1 (dose: (500 nΜ for 24 h)
PAP-1	**in vivo:** MitoPark, MPTP, and αSyn_PFF_ PD mouse model (dose: daily with 40 mg/kg, intraperitoneally) **in vitro:** primary microglia from neonatal mica (dose: 1 µL)
**Pharmacological drugs**	
AD-16	**in vivo:** 6-hydroxydopamine (6-OHDA) mouse model of PD (dose: 1 mg/kg of AD-16 administered intraperitoneally)
NDGA	**in vivo:** transient MCAO mouse model (dose: 5 mg/kg or 20 mg/kg injected intraperitoneally at 3 h, 6 h, 24 h, and 48 h after middle cerebral artery occlusion). **in vitro:** lipopolysaccharide (LPS)-primed NLRP3 inflammasome model in primary mouse microglia cells and human primary monocytes (inhibitory concentration of 5.958 μM to 10 μM).
BA	**in vivo:** MCAO mouse model (dose: BA (10 mg/kg) intraperitoneally injected 5 h after middle cerebral artery occlusion). **in vitro:** LPS-stimulated pro-inflammatory responses of primary microglia BV2 cells (dose: high-dose 5 or 10 μM).
H-151	**in vivo:** transient MCAO mouse model (dose: 10 mg/kg administered intraperitoneally). **in vitro:** primary microglia isolated from mouse pups on post-natal days 1–3 (dose: 1 μmol/L for 48 h).
**Cytokines and peptides**	
IL-10	**in vivo:** PD mouse model generated following human α-synuclein (LV:SNCA) lentiviral vector injections in the SN (dose: No precise LV:μgIL10 dosage was mentioned, but a volume of 1.5 µL of LVs (5 × 10^9^ TU/mL) was administered to each hemisphere at a controlled flow rate of 0.25 µL/min).
MANF	**in vivo:** rotenone-induced PD mouse model (dose: 0.5 μL of AAV8-MANF (1 × 10^13^ v.g/mL) injected into the SN at a rate of 1 μL/min). **in vitro:** BV2 cells (dose: 250, 500 ng/mL).
RhFGF21	**in vivo:** MCAO mouse model (dose: 1.5 mg/kg for 7 consecutive days) **in vitro:** primary rat microglia culture and BV2 cells (dose: 100 nM).
**Plant-based compounds**	
Cedrol	**in vivo:** MCAO mouse model (dose: 40 mg/kg intragastrically for 3 days) **in vitro:** primary microglia isolated from mouse pups on post-natal day 1 (dose: 5–50 μM for 2 h)
Ligustilide	**in vivo:** transient MCAO mouse model (dose: 20 and 60 mg/kg was administered via oral gavage at 1, 24, and 48 h post-ischemia/reperfusion) **in vitro:** primary microglia isolated from Kunming mouse pups on post-natal days 3 (dose: 2.5, 5, 10 μM for 2 h).
FT	**in vivo:** spontaneous hypertensive rat (dose: 10.8 g/kg given by gavage once daily for 5 weeks)
KAE	**in vivo:** - 6-hydroxydopamine (6-OHDA) mouse model of PD (dose: 50 mg/kg intragastrically administred 30 consecutive days). - Transient MCAO mouse model (dose: delivered by intragastric at doses of 25, 50 and 100 mg/kg body weight per day for 7 days). **in vitro:** LPS-treated BV2 microglial cells (dose: 90 μM KAE for 2 h prior to LPS treatment).
Vanadium-curcumin	**in vitro:** primary mixed glia and neuronal cells isolated from rat pups on post-natal days 6–8 (dose: 2 µM to co-cultures for 30 min).

## Data Availability

Not applicable.

## References

[1] Sweeney M.D., Zhao Z., Montagne A., Nelson A.R., Zlokovic B.V. (2019). Blood-Brain Barrier: From Physiology to Disease and Back. Physiol. Rev..

[2] Rhea E.M., Banks W.A. (2019). Role of the Blood-Brain Barrier in Central Nervous System Insulin Resistance. Front. Neurosci..

[3] Yu X., Ji C., Shao A. (2020). Neurovascular Unit Dysfunction and Neurodegenerative Disorders. Front. Neurosci..

[4] Zlokovic B.V. (2011). Neurovascular Pathways to Neurodegeneration in Alzheimer’s Disease and Other Disorders. Nat. Rev. Neurosci..

[5] Knox E.G., Aburto M.R., Clarke G., Cryan J.F., O’Driscoll C.M. (2022). The Blood-Brain Barrier in Aging and Neurodegeneration. Mol. Psychiatry.

[6] Sun R., Jiang H. (2024). Border-Associated Macrophages in the Central Nervous System. J. Neuroinflamm..

[7] Adamu A., Li S., Gao F., Xue G. (2024). The Role of Neuroinflammation in Neurodegenerative Diseases: Current Understanding and Future Therapeutic Targets. Front. Aging Neurosci..

[8] Patabendige A., Janigro D. (2023). The Role of the Blood–Brain Barrier during Neurological Disease and Infection. Biochem. Soc. Trans..

[9] Zhang Z., Duan Z., Cui Y. (2023). CD8+ T Cells in Brain Injury and Neurodegeneration. Front. Cell Neurosci..

[10] Zhang J., Zhang Y., Wang J., Xia Y., Zhang J., Chen L. (2024). Recent Advances in Alzheimer’s Disease: Mechanisms, Clinical Trials and New Drug Development Strategies. Signal Transduct. Target. Ther..

[11] Qin J., Ma Z., Chen X., Shu S. (2023). Microglia Activation in Central Nervous System Disorders: A Review of Recent Mechanistic Investigations and Development Efforts. Front. Neurol..

[12] Borst K., Dumas A.A., Prinz M. (2021). Microglia: Immune and Non-Immune Functions. Immunity.

[13] Machhi J., Kevadiya B.D., Muhammad I.K., Herskovitz J., Olson K.E., Mosley R.L., Gendelman H.E. (2020). Harnessing Regulatory T Cell Neuroprotective Activities for Treatment of Neurodegenerative Disorders. Mol. Neurodegener..

[14] Koh C.-H., Lee S., Kwak M., Kim B.-S., Chung Y. (2023). CD8 T-Cell Subsets: Heterogeneity, Functions, and Therapeutic Potential. Exp. Mol. Med..

[15] Martins-Ferreira R., Calafell-Segura J., Leal B., Rodríguez-Ubreva J., Martínez-Saez E., Mereu E., Pinho E Costa P., Laguna A., Ballestar E. (2025). The Human Microglia Atlas (HuMicA) Unravels Changes in Disease-Associated Microglia Subsets across Neurodegenerative Conditions. Nat. Commun..

[16] Valiukas Z., Tangalakis K., Apostolopoulos V., Feehan J. (2025). Microglial Activation States and Their Implications for Alzheimer’s Disease. J. Prev. Alzheimers Dis..

[17] Zheng Q., Wang X. (2025). Alzheimer’s Disease: Insights into Pathology, Molecular Mechanisms, and Therapy. Protein Cell.

[18] DeKosky S.T., Scheff S.W. (1990). Synapse Loss in Frontal Cortex Biopsies in Alzheimer’s Disease: Correlation with Cognitive Severity. Ann. Neurol..

[19] Terry R.D., Masliah E., Salmon D.P., Butters N., DeTeresa R., Hill R., Hansen L.A., Katzman R. (1991). Physical Basis of Cognitive Alterations in Alzheimer’s Disease: Synapse Loss Is the Major Correlate of Cognitive Impairment. Ann. Neurol..

[20] Hong S., Beja-Glasser V.F., Nfonoyim B.M., Frouin A., Li S., Ramakrishnan S., Merry K.M., Shi Q., Rosenthal A., Barres B.A. (2016). Complement and Microglia Mediate Early Synapse Loss in Alzheimer Mouse Models. Science.

[21] Ni W., Ding J., Gong P., Tan X., Li J. (2025). Inhibition of Kv1.1 Channels Ameliorates Cu(II)-Induced Microglial Activation and Cognitive Impairment in Mice. Neurochem. Int..

[22] Chen L.-L., Fan Y.-G., Zhao L.-X., Zhang Q., Wang Z.-Y. (2023). The Metal Ion Hypothesis of Alzheimer’s Disease and the Anti-Neuroinflammatory Effect of Metal Chelators. Bioorg. Chem..

[23] Ejaz H.W., Wang W., Lang M. (2020). Copper Toxicity Links to Pathogenesis of Alzheimer’s Disease and Therapeutics Approaches. Int. J. Mol. Sci..

[24] Lim S.L., Rodriguez-Ortiz C.J., Hsu H.-W., Wu J., Zumkehr J., Kilian J., Vidal J., Ayata P., Kitazawa M. (2020). Chronic Copper Exposure Directs Microglia towards Degenerative Expression Signatures in Wild-Type and J20 Mouse Model of Alzheimer’s Disease. J. Trace Elem. Med. Biol..

[25] Fremuth L.E., Hu H., van de Vlekkert D., Annunziata I., Weesner J.A., d′Azzo A. (2025). Neuraminidase 1 Regulates Neuropathogenesis by Governing the Cellular State of Microglia via Modulation of Trem2 Sialylation. Cell Rep..

[26] Sha X., Lin J., Wu K., Lu J., Yu Z. (2025). The TRPV1-PKM2-SREBP1 Axis Maintains Microglial Lipid Homeostasis in Alzheimer’s Disease. Cell Death Dis..

[27] Mhyre T.R., Boyd J.T., Hamill R.W., Maguire-Zeiss K.A. (2012). Parkinson’s Disease. Protein Aggregation and Fibrillogenesis in Cerebral and Systemic Amyloid Disease. Subcellular Biochemistry.

[28] Balestrino R., Schapira A.H.V. (2020). Parkinson Disease. Eur. J. Neurol..

[29] Su X., Federoff H.J., Maguire-Zeiss K.A. (2009). Mutant Alpha-Synuclein Overexpression Mediates Early Proinflammatory Activity. Neurotox. Res..

[30] Zhang Z., Niu K., Huang T., Guo J., Xarbat G., Gong X., Gao Y., Liu F., Cheng S., Su W. (2025). Microglia Depletion Reduces Neurodegeneration and Remodels Extracellular Matrix in a Mouse Parkinson’s Disease Model Triggered by α-Synuclein Overexpression. NPJ Park. Dis..

[31] Torrente D., Su E.J., Citalán-Madrid A.F., Schielke G.P., Magaoay D., Warnock M., Stevenson T., Mann K., Lesept F., Delétage N. (2025). The Interaction of TPA with NMDAR1 Drives Neuroinflammation and Neurodegeneration in α-Synuclein-Mediated Neurotoxicity. J. Neuroinflamm..

[32] Yu H., Chang Q., Sun T., He X., Wen L., An J., Feng J., Zhao Y. (2023). Metabolic Reprogramming and Polarization of Microglia in Parkinson’s Disease: Role of Inflammasome and Iron. Ageing Res. Rev..

[33] Geng L., Gao W., Saiyin H., Li Y., Zeng Y., Zhang Z., Li X., Liu Z., Gao Q., An P. (2023). MLKL Deficiency Alleviates Neuroinflammation and Motor Deficits in the α-Synuclein Transgenic Mouse Model of Parkinson’s Disease. Mol. Neurodegener..

[34] Zhou Q., Zhang Y., Lu L., Zhang H., Zhao C., Pu Y., Yin L. (2022). Copper Induces Microglia-Mediated Neuroinflammation through ROS/NF-ΚB Pathway and Mitophagy Disorder. Food Chem. Toxicol..

[35] Galindo A.N., Frey Rubio D.A., Hettiaratchi M.H. (2024). Biomaterial Strategies for Regulating the Neuroinflammatory Response. Mater. Adv..

[36] Xie M., Hao Y., Feng L., Wang T., Yao M., Li H., Ma D., Feng J. (2023). Neutrophil Heterogeneity and Its Roles in the Inflammatory Network after Ischemic Stroke. Curr. Neuropharmacol..

[37] Kunz A., Iadecola C. (2008). Chapter 14 Cerebral Vascular Dysregulation in the Ischemic Brain. Handbook of Clinical Neurology.

[38] Santos Samary C., Pelosi P., Leme Silva P., Rieken Macedo Rocco P. (2016). Immunomodulation after Ischemic Stroke: Potential Mechanisms and Implications for Therapy. Crit. Care.

[39] Chamorro Á., Meisel A., Planas A.M., Urra X., van de Beek D., Veltkamp R. (2012). The Immunology of Acute Stroke. Nat. Rev. Neurol..

[40] Dziedzic T. (2015). Systemic Inflammation as a Therapeutic Target in Acute Ischemic Stroke. Expert. Rev. Neurother..

[41] Zhang Y., Guo Y., Li R., Huang T., Li Y., Xie W., Chen C., Chen W., Wan J., Yu W. (2023). Novel CH25H+ and OASL+ Microglia Subclusters Play Distinct Roles in Cerebral Ischemic Stroke. J. Neuroinflamm..

[42] Yang T., Guo R., Zhang F. (2019). Brain Perivascular Macrophages: Recent Advances and Implications in Health and Diseases. CNS Neurosci. Ther..

[43] Jeong H.-W., Diéguez-Hurtado R., Arf H., Song J., Park H., Kruse K., Sorokin L., Adams R.H. (2022). Single-Cell Transcriptomics Reveals Functionally Specialized Vascular Endothelium in Brain. eLife.

[44] Karam M., Janbon H., Malkinson G., Brunet I. (2022). Heterogeneity and Developmental Dynamics of LYVE-1 Perivascular Macrophages Distribution in the Mouse Brain. J. Cereb. Blood Flow. Metab..

[45] Drieu A., Du S., Storck S.E., Rustenhoven J., Papadopoulos Z., Dykstra T., Zhong F., Kim K., Blackburn S., Mamuladze T. (2022). Parenchymal Border Macrophages Regulate the Flow Dynamics of the Cerebrospinal Fluid. Nature.

[46] Hu M., Li T., Ma X., Liu S., Li C., Huang Z., Lin Y., Wu R., Wang S., Lu D. (2023). Macrophage Lineage Cells-Derived Migrasomes Activate Complement-Dependent Blood-Brain Barrier Damage in Cerebral Amyloid Angiopathy Mouse Model. Nat. Commun..

[47] Taylor X., Clark I.M., Fitzgerald G.J., Oluoch H., Hole J.T., DeMattos R.B., Wang Y., Pan F. (2023). Amyloid-β (Aβ) Immunotherapy Induced Microhemorrhages Are Associated with Activated Perivascular Macrophages and Peripheral Monocyte Recruitment in Alzheimer’s Disease Mice. Mol. Neurodegener..

[48] Park L., Uekawa K., Garcia-Bonilla L., Koizumi K., Murphy M., Pistik R., Younkin L., Younkin S., Zhou P., Carlson G. (2017). Brain Perivascular Macrophages Initiate the Neurovascular Dysfunction of Alzheimer Aβ Peptides. Circ. Res..

[49] Faraco G., Sugiyama Y., Lane D., Garcia-Bonilla L., Chang H., Santisteban M.M., Racchumi G., Murphy M., Van Rooijen N., Anrather J. (2016). Perivascular Macrophages Mediate the Neurovascular and Cognitive Dysfunction Associated with Hypertension. J. Clin. Investig..

[50] Santisteban M.M., Ahn S.J., Lane D., Faraco G., Garcia-Bonilla L., Racchumi G., Poon C., Schaeffer S., Segarra S.G., Körbelin J. (2020). Endothelium-Macrophage Crosstalk Mediates Blood-Brain Barrier Dysfunction in Hypertension. Hypertension.

[51] Drieu A., Du S., Kipnis M., Bosch M.E., Herz J., Lee C., Jiang H., Manis M., Ulrich J.D., Kipnis J. (2023). Parenchymal Border Macrophages Regulate Tau Pathology and Tau-Mediated Neurodegeneration. Life Sci. Alliance.

[52] De Schepper S., Ge J.Z., Crowley G., Ferreira L.S.S., Garceau D., Toomey C.E., Sokolova D., Rueda-Carrasco J., Shin S.-H., Kim J.-S. (2023). Perivascular Cells Induce Microglial Phagocytic States and Synaptic Engulfment via SPP1 in Mouse Models of Alzheimer’s Disease. Nat. Neurosci..

[53] Schonhoff A.M., Figge D.A., Williams G.P., Jurkuvenaite A., Gallups N.J., Childers G.M., Webster J.M., Standaert D.G., Goldman J.E., Harms A.S. (2023). Border-Associated Macrophages Mediate the Neuroinflammatory Response in an Alpha-Synuclein Model of Parkinson Disease. Nat. Commun..

[54] Pedragosa J., Salas-Perdomo A., Gallizioli M., Cugota R., Miró-Mur F., Briansó F., Justicia C., Pérez-Asensio F., Marquez-Kisinousky L., Urra X. (2018). CNS-Border Associated Macrophages Respond to Acute Ischemic Stroke Attracting Granulocytes and Promoting Vascular Leakage. Acta Neuropathol. Commun..

[55] Alshoubaki Y.K., Nayer B., Das S., Martino M.M. (2022). Modulation of the Activity of Stem and Progenitor Cells by Immune Cells. Stem Cells Transl. Med..

[56] Liu H., Zhao Y., Xie A., Kim T.-Y., Terentyeva R., Liu M., Shi G., Feng F., Choi B.-R., Terentyev D. (2021). Interleukin-1β, Oxidative Stress, and Abnormal Calcium Handling Mediate Diabetic Arrhythmic Risk. JACC Basic. Transl. Sci..

[57] Soung A.L., Davé V.A., Garber C., Tycksen E.D., Vollmer L.L., Klein R.S. (2022). IL-1 Reprogramming of Adult Neural Stem Cells Limits Neurocognitive Recovery after Viral Encephalitis by Maintaining a Proinflammatory State. Brain Behav. Immun..

[58] Arranz L., Arriero M.d.M., Villatoro A. (2017). Interleukin-1β as Emerging Therapeutic Target in Hematological Malignancies and Potentially in Their Complications. Blood Rev..

[59] Carrero R., Cerrada I., Lledó E., Dopazo J., García-García F., Rubio M.-P., Trigueros C., Dorronsoro A., Ruiz-Sauri A., Montero J.A. (2012). IL1β Induces Mesenchymal Stem Cells Migration and Leucocyte Chemotaxis Through NF-ΚB. Stem Cell Rev. Rep..

[60] Sullivan C.B., Porter R.M., Evans C.H., Ritter T., Shaw G., Barry F., Murphy J.M. (2014). TNFα and IL-1β Influence the Differentiation and Migration of Murine MSCs Independently of the NF-ΚB Pathway. Stem Cell Res. Ther..

[61] Colombini A., Libonati F., Cangelosi D., Lopa S., De Luca P., Coviello D.A., Moretti M., de Girolamo L. (2022). Inflammatory Priming with IL-1β Promotes the Immunomodulatory Behavior of Adipose Derived Stem Cells. Front. Bioeng. Biotechnol..

[62] Zeng Y.-X., Chou K.-Y., Hwang J.-J., Wang H.-S. (2023). The Effects of IL-1β Stimulated Human Umbilical Cord Mesenchymal Stem Cells on Polarization and Apoptosis of Macrophages in Rheumatoid Arthritis. Sci. Rep..

[63] Park S.-Y., Kang M.-J., Han J.-S. (2018). Interleukin-1 Beta Promotes Neuronal Differentiation through the Wnt5a/RhoA/JNK Pathway in Cortical Neural Precursor Cells. Mol. Brain.

[64] Williams J.L., Patel J.R., Daniels B.P., Klein R.S. (2014). Targeting CXCR7/ACKR3 as a Therapeutic Strategy to Promote Remyelination in the Adult Central Nervous System. J. Exp. Med..

[65] McCandless E.E., Piccio L., Woerner B.M., Schmidt R.E., Rubin J.B., Cross A.H., Klein R.S. (2008). Pathological Expression of CXCL12 at the Blood-Brain Barrier Correlates with Severity of Multiple Sclerosis. Am. J. Pathol..

[66] Turrin N.P., Rivest S. (2006). Tumor Necrosis Factor α But Not Interleukin 1β Mediates Neuroprotection in Response to Acute Nitric Oxide Excitotoxicity. J. Neurosci..

[67] Fontaine V., Mohand-Said S., Hanoteau N., Fuchs C., Pfizenmaier K., Eisel U. (2002). Neurodegenerative and Neuroprotective Effects of Tumor Necrosis Factor (TNF) in Retinal Ischemia: Opposite Roles of TNF Receptor 1 and TNF Receptor 2. J. Neurosci..

[68] Belenguer G., Duart-Abadia P., Jordán-Pla A., Domingo-Muelas A., Blasco-Chamarro L., Ferrón S.R., Morante-Redolat J.M., Fariñas I. (2021). Adult Neural Stem Cells Are Alerted by Systemic Inflammation through TNF-α Receptor Signaling. Cell Stem Cell.

[69] Dorronsoro A., Ferrin I., Salcedo J.M., Jakobsson E., Fernández-Rueda J., Lang V., Sepulveda P., Fechter K., Pennington D., Trigueros C. (2014). Human Mesenchymal Stromal Cells Modulate T-cell Responses through TNF-α-mediated Activation of NF-κB. Eur. J. Immunol..

[70] Wang J., Xu L., Peng D., Zhu Y., Gu Z., Yao Y., Li H., Cao X., Fu C., Zheng M. (2023). IFN-γ-STAT1-Mediated CD8+ T-Cell-Neural Stem Cell Cross Talk Controls Astrogliogenesis after Spinal Cord Injury. Inflamm. Regen..

[71] Hu S., Rotschafer J.H., Lokensgard J.R., Cheeran M.C.-J. (2014). Activated CD8+ T Lymphocytes Inhibit Neural Stem/Progenitor Cell Proliferation: Role of Interferon-Gamma. PLoS ONE.

[72] Rotschafer J.H., Hu S., Little M., Erickson M., Low W.C., Cheeran M.C.J. (2013). Modulation of Neural Stem/Progenitor Cell Proliferation during Experimental Herpes Simplex Encephalitis Is Mediated by Differential FGF-2 Expression in the Adult Brain. Neurobiol. Dis..

[73] Kummer K.K., Zeidler M., Kalpachidou T., Kress M. (2021). Role of IL-6 in the Regulation of Neuronal Development, Survival and Function. Cytokine.

[74] Kerkis I., Silva Á.P.d., Araldi R.P. (2024). The Impact of Interleukin-6 (IL-6) and Mesenchymal Stem Cell-Derived IL-6 on Neurological Conditions. Front. Immunol..

[75] Hwang I., Tang D., Paik J. (2021). Oxidative Stress Sensing and Response in Neural Stem Cell Fate. Free Radic. Biol. Med..

[76] Hu Q., Khanna P., Ee Wong B.S., Lin Heng Z.S., Subhramanyam C.S., Thanga L.Z., Sing Tan S.W., Baeg G.H. (2018). Oxidative Stress Promotes Exit from the Stem Cell State and Spontaneous Neuronal Differentiation. Oncotarget.

[77] Liguori C., Stefani A., Sancesario G., Sancesario G.M., Marciani M.G., Pierantozzi M. (2015). CSF Lactate Levels, τ Proteins, Cognitive Decline: A Dynamic Relationship in Alzheimer’s Disease. J. Neurol. Neurosurg. Psychiatry.

[78] Schirinzi T., Di Lazzaro G., Sancesario G.M., Summa S., Petrucci S., Colona V.L., Bernardini S., Pierantozzi M., Stefani A., Mercuri N.B. (2020). Young-Onset and Late-Onset Parkinson’s Disease Exhibit a Different Profile of Fluid Biomarkers and Clinical Features. Neurobiol. Aging.

[79] Qin Q., Wang D., Qu Y., Li J., An K., Mao Z., Li J., Xiong Y., Min Z., Xue Z. (2025). Enhanced Glycolysis-Derived Lactate Promotes Microglial Activation in Parkinson’s Disease via Histone Lactylation. NPJ Park. Dis..

[80] Pan R.-Y., He L., Zhang J., Liu X., Liao Y., Gao J., Liao Y., Yan Y., Li Q., Zhou X. (2022). Positive Feedback Regulation of Microglial Glucose Metabolism by Histone H4 Lysine 12 Lactylation in Alzheimer’s Disease. Cell Metab..

[81] Gu R., Zhang F., Chen G., Han C., Liu J., Ren Z., Zhu Y., Waddington J.L., Zheng L.T., Zhen X. (2017). Clk1 Deficiency Promotes Neuroinflammation and Subsequent Dopaminergic Cell Death through Regulation of Microglial Metabolic Reprogramming. Brain Behav. Immun..

[82] Luo G., Wang X., Cui Y., Cao Y., Zhao Z., Zhang J. (2021). Metabolic Reprogramming Mediates Hippocampal Microglial M1 Polarization in Response to Surgical Trauma Causing Perioperative Neurocognitive Disorders. J. Neuroinflamm..

[83] Si W.-Y., Yang C.-L., Wei S.-L., Du T., Li L.-K., Dong J., Zhou Y., Li H., Zhang P., Liu Q.-J. (2024). Therapeutic Potential of Microglial SMEK1 in Regulating H3K9 Lactylation in Cerebral Ischemia-Reperfusion. Commun. Biol..

[84] Castillo X., Rosafio K., Wyss M.T., Drandarov K., Buck A., Pellerin L., Weber B., Hirt L. (2015). A Probable Dual Mode of Action for Both L- and D-Lactate Neuroprotection in Cerebral Ischemia. J. Cereb. Blood Flow. Metab..

[85] Berthet C., Lei H., Thevenet J., Gruetter R., Magistretti P.J., Hirt L. (2009). Neuroprotective Role of Lactate after Cerebral Ischemia. J. Cereb. Blood Flow. Metab..

[86] Zhang Y., Zhang S., Yang L., Zhang Y., Cheng Y., Jia P., Lv Y., Wang K., Fan P., Zhang P. (2025). Lactate Modulates Microglial Inflammatory Responses through HIF-1α-Mediated CCL7 Signaling after Cerebral Ischemia in Mice. Int. Immunopharmacol..

[87] Gentili V., Schiuma G., Dilliraj L.N., Beltrami S., Rizzo S., Lara D., Giovannini P.P., Marti M., Bortolotti D., Trapella C. (2024). DAG-MAG-ΒHB: A Novel Ketone Diester Modulates NLRP3 Inflammasome Activation in Microglial Cells in Response to Beta-Amyloid and Low Glucose AD-like Conditions. Nutrients.

[88] Zhang J., Zhang Y., Liu L., Zhang M., Zhang X., Deng J., Zhao F., Lin Q., Zheng X., Fu B. (2025). Chemerin-9 Is Neuroprotective in APP/PS1 Transgenic Mice by Inhibiting NLRP3 Inflammasome and Promoting Microglial Clearance of Aβ. J. Neuroinflamm..

[89] Sarkar S., Nguyen H.M., Malovic E., Luo J., Langley M., Palanisamy B.N., Singh N., Manne S., Neal M., Gabrielle M. (2020). Kv1.3 Modulates Neuroinflammation and Neurodegeneration in Parkinson’s Disease. J. Clin. Investig..

[90] Peng D., Xu S., Zou T., Wang Y., Ouyang W., Zhang Y., Dong C., Li D., Guo J., Shen Q. (2023). Safety, Tolerability, Pharmacokinetics and Effects of Diet on AD16, a Novel Neuroinflammatory Inhibitor for Alzheimer’s Disease: A Randomized Phase 1 Study. BMC Med..

[91] Huang Z., Luo Z., Ovcjak A., Wan J., Chen N., Hu W., Sun H.-S., Feng Z.-P. (2022). AD-16 Protects Against Hypoxic-Ischemic Brain Injury by Inhibiting Neuroinflammation. Neurosci. Bull..

[92] Zhang L., Zhao G., Luo Z., Yu Z., Liu G., Su G., Tang X., Yuan Z., Huang C., Sun H.-S. (2024). AD16 Attenuates Neuroinflammation Induced by Cerebral Ischemia through Down-Regulating Astrocytes A1 Polarization. Biomed. Pharmacother..

[93] Bianchetti M.E., Ferreira A.F.F., Britto L.R.G. (2024). Inhibition of Neuroinflammation by GIBH-130 (AD-16) Reduces Neurodegeneration, Motor Deficits, and Proinflammatory Cytokines in a Hemiparkinsonian Model. Front. Neuroanat..

[94] Guan X., Zhu S., Song J., Liu K., Liu M., Xie L., Wang Y., Wu J., Xu X., Pang T. (2024). Microglial CMPK2 Promotes Neuroinflammation and Brain Injury after Ischemic Stroke. Cell Rep. Med..

[95] Smyth P., Sasiwachirangkul J., Williams R., Scott C.J. (2022). Cathepsin S (CTSS) Activity in Health and Disease—A Treasure Trove of Untapped Clinical Potential. Mol. Asp. Med..

[96] Pišlar A., Kos J. (2014). Cysteine Cathepsins in Neurological Disorders. Mol. Neurobiol..

[97] Xie L., Zhang S., Huang L., Peng Z., Lu H., He Q., Chen R., Hu L., Wang B., Sun B. (2023). Single-Cell RNA Sequencing of Peripheral Blood Reveals That Monocytes with High Cathepsin S Expression Aggravate Cerebral Ischemia–Reperfusion Injury. Brain Behav. Immun..

[98] Zhang Y., Yang L., Gan Y., Zhao C., Zhou C., Chen J., Yin Y., Xia S., Yang H., Bao X. (2025). Benzydamine Attenuates Microglia-Mediated Neuroinflammation and Ischemic Brain Injury by Targeting Cathepsin s. Int. Immunopharmacol..

[99] Kong L., Xu P., Shen N., Li W., Li R., Tao C., Wang G., Zhang Y., Sun W., Hu W. (2024). STING Orchestrates Microglia Polarization via Interaction with LC3 in Autophagy after Ischemia. Cell Death Dis..

[100] Liu Z., Qin Q., Wang S., Kang X., Liu Y., Wei L., Lu Z., Cai W., Hu M. (2024). STING Activation in Macrophages and Microglia Drives Poststroke Inflammation: Implications for Neuroinflammatory Mechanisms and Therapeutic Interventions. CNS Neurosci. Ther..

[101] Sui H., Sun Z., Liu C., Xi H. (2025). Ferritinophagy Promotes Microglia Ferroptosis to Aggravate Neuroinflammation Induced by Cerebral Ischemia-Reperfusion Injury via Activation of the CGAS-STING Signaling Pathway. Neurochem. Int..

[102] Bido S., Nannoni M., Muggeo S., Gambarè D., Ruffini G., Bellini E., Passeri L., Iaia S., Luoni M., Provinciali M. (2024). Microglia-Specific *IL-10* Gene Delivery Inhibits Neuroinflammation and Neurodegeneration in a Mouse Model of Parkinson’s Disease. Sci. Transl. Med..

[103] Zhou K.-G., Huang Y.-B., Zhu Z.-W., Jiang M., Jin L.-J., Guan Q., Tian L.-L., Zhang J.-X. (2025). Mesencephalic Astrocyte-Derived Neurotrophic Factor Inhibits Neuroinflammation through Autophagy-Mediated α-Synuclein Degradation. Arch. Gerontol. Geriatr..

[104] Wang D., Liu F., Zhu L., Lin P., Han F., Wang X., Tan X., Lin L., Xiong Y. (2020). FGF21 Alleviates Neuroinflammation Following Ischemic Stroke by Modulating the Temporal and Spatial Dynamics of Microglia/Macrophages. J. Neuroinflamm..

[105] Yang X., Hui Q., Yu B., Huang Z., Zhou P., Wang P., Wang Z., Pang S., Li J., Wang H. (2018). Design and Evaluation of Lyophilized Fibroblast Growth Factor 21 and Its Protection against Ischemia Cerebral Injury. Bioconjug. Chem..

[106] Bi Y., Xie Z., Cao X., Ni H., Xia S., Bao X., Huang Q., Xu Y., Zhang Q. (2024). Cedrol Attenuates Acute Ischemic Injury through Inhibition of Microglia-Associated Neuroinflammation via ERβ-NF-ΚB Signaling Pathways. Brain Res. Bull..

[107] Chen X., Shen J., Zhao J., Guan J., Li W., Xie Q., Zhao Y. (2020). Cedrol Attenuates Collagen-Induced Arthritis in Mice and Modulates the Inflammatory Response in LPS-Mediated Fibroblast-like Synoviocytes. Food Funct..

[108] Gao J., Su G., Liu J., Song J., Chen W., Chai M., Xie X., Wang M., Liu J., Zhang Z. (2024). A Novel Compound Ligusticum Cycloprolactam Alleviates Neuroinflammation After Ischemic Stroke via the FPR1/NLRP3 Signaling Axis. CNS Neurosci. Ther..

[109] Sun X., Wang S., Sheng H., Lv X., Li J., Han B., Wang S., Liu K., Zhang C., Zhang W. (2023). Study on the Mechanism of Stir-Fried Fructus Tribuli in Enhancing the Essential Hypertension Treatment by an Integrated “Spectrum-Effect Relationship-Network Pharmacology-Metabolomics” Strategy. Biomed. Pharmacother..

[110] Yan A., Liu Z., Song L., Wang X., Zhang Y., Wu N., Lin J., Liu Y., Liu Z. (2019). Idebenone Alleviates Neuroinflammation and Modulates Microglial Polarization in LPS-Stimulated BV2 Cells and MPTP-Induced Parkinson’s Disease Mice. Front. Cell Neurosci..

[111] Cai M., Zhuang W., Lv E., Liu Z., Wang Y., Zhang W., Fu W. (2022). Kaemperfol Alleviates Pyroptosis and Microglia-Mediated Neuroinflammation in Parkinson’s Disease via Inhibiting P38MAPK/NF-ΚB Signaling Pathway. Neurochem. Int..

[112] Zhao Y., Kuca K., Wu W., Wang X., Nepovimova E., Musilek K., Wu Q. (2022). Hypothesis: JNK Signaling Is a Therapeutic Target of Neurodegenerative Diseases. Alzheimer’s Dement..

[113] Li W.-H., Cheng X., Yang Y.-L., Liu M., Zhang S.-S., Wang Y.-H., Du G.-H. (2019). Kaempferol Attenuates Neuroinflammation and Blood Brain Barrier Dysfunction to Improve Neurological Deficits in Cerebral Ischemia/Reperfusion Rats. Brain Res..

[114] Katsipis G., Lavrentiadou S.N., Geromichalos G.D., Tsantarliotou M.P., Halevas E., Litsardakis G., Pantazaki A.A. (2024). Evaluation of the Anti-Amyloid and Anti-Inflammatory Properties of a Novel Vanadium(IV)-Curcumin Complex in Lipopolysaccharides-Stimulated Primary Rat Neuron-Microglia Mixed Cultures. Int. J. Mol. Sci..

[115] Lin J., Wu Y., Liu G., Cui R., Xu Y. (2024). Advances of Ultrasound in Tumor Immunotherapy. Int. Immunopharmacol..

[116] Chen C.-M., Wu C.-T., Yang T.-H., Liu S.-H., Yang F.-Y. (2018). Preventive Effect of Low Intensity Pulsed Ultrasound against Experimental Cerebral Ischemia/Reperfusion Injury via Apoptosis Reduction and Brain-Derived Neurotrophic Factor Induction. Sci. Rep..

[117] Li Y., Teng X., Yang C., Wang Y., Wang L., Dai Y., Sun H., Li J. (2021). Ultrasound Controlled Anti-Inflammatory Polarization of Platelet Decorated Microglia for Targeted Ischemic Stroke Therapy. Angew. Chem. Int. Ed..

[118] Hong Z., Zuo Z., Zhao Y., Ai Y., Zhang L., Li L., He X., Luo J., Xu J., Yang X. (2025). Transcranial Focused Ultrasound Stimulation Alleviates NLRP3-Related Neuroinflammation Induced by Ischemic Stroke via Regulation of the Nespas/MiR-383-3p/SHP2 Pathway. Int. Immunopharmacol..

[119] Wang J., Li G., Deng L., Mamtilahun M., Jiang L., Qiu W., Zheng H., Sun J., Xie Q., Yang G.-Y. (2021). Transcranial Focused Ultrasound Stimulation Improves Neurorehabilitation after Middle Cerebral Artery Occlusion in Mice. Aging Dis..

[120] Wattananit S., Tornero D., Graubardt N., Memanishvili T., Monni E., Tatarishvili J., Miskinyte G., Ge R., Ahlenius H., Lindvall O. (2016). Monocyte-Derived Macrophages Contribute to Spontaneous Long-Term Functional Recovery after Stroke in Mice. J. Neurosci..

[121] Noronha N.d.C., Mizukami A., Caliári-Oliveira C., Cominal J.G., Rocha J.L.M., Covas D.T., Swiech K., Malmegrim K.C.R. (2019). Priming Approaches to Improve the Efficacy of Mesenchymal Stromal Cell-Based Therapies. Stem Cell Res. Ther..

[122] Ge R., Tornero D., Hirota M., Monni E., Laterza C., Lindvall O., Kokaia Z. (2017). Choroid Plexus-Cerebrospinal Fluid Route for Monocyte-Derived Macrophages after Stroke. J. Neuroinflamm..

[123] Tolstova T., Dotsenko E., Luzgina N., Rusanov A. (2024). Preconditioning of Mesenchymal Stem Cells Enhances the Neuroprotective Effects of Their Conditioned Medium in an Alzheimer’s Disease In Vitro Model. Biomedicines.

[124] Li H., Yahaya B.H., Ng W.H., Yusoff N.M., Lin J. (2019). Conditioned Medium of Human Menstrual Blood-Derived Endometrial Stem Cells Protects Against MPP+-Induced Cytotoxicity in Vitro. Front. Mol. Neurosci..

[125] Li H., Wei J., Zhang Z., Li J., Ma Y., Zhang P., Lin J. (2023). Menstrual Blood-Derived Endometrial Stem Cells Alleviate Neuroinflammation by Modulating M1/M2 Polarization in Cell and Rat Parkinson’s Disease Models. Stem Cell Res. Ther..

[126] Fričová D., Korchak J.A., Zubair A.C. (2020). Challenges and Translational Considerations of Mesenchymal Stem/Stromal Cell Therapy for Parkinson’s Disease. NPJ Regen. Med..

[127] Mehrabadi S., Motevaseli E., Sadr S.S., Moradbeygi K. (2020). Hypoxic-Conditioned Medium from Adipose Tissue Mesenchymal Stem Cells Improved Neuroinflammation through Alternation of Toll like Receptor (TLR) 2 and TLR4 Expression in Model of Alzheimer’s Disease Rats. Behav. Brain Res..

[128] Yamazaki H., Jin Y., Tsuchiya A., Kanno T., Nishizaki T. (2015). Adipose-Derived Stem Cell-Conditioned Medium Ameliorates Antidepression-Related Behaviors in the Mouse Model of Alzheimer’s Disease. Neurosci. Lett..

[129] Wan Z., Mah D., Simtchouk S., Kluftinger A., Little J. (2015). Human Adipose Tissue Conditioned Media from Lean Subjects Is Protective against H2O2 Induced Neurotoxicity in Human SH-SY5Y Neuronal Cells. Int. J. Mol. Sci..

[130] Alidoust L., Akhoondian M., Atefi A.H., Keivanlou M.-H., Hedayati Ch M., Jafari A. (2023). Stem Cell-Conditioned Medium Is a Promising Treatment for Alzheimer’s Disease. Behav. Brain Res..

[131] Shin J.Y., Kim D.-Y., Lee J., Shin Y.J., Kim Y.S., Lee P.H. (2022). Priming Mesenchymal Stem Cells with α-Synuclein Enhances Neuroprotective Properties through Induction of Autophagy in Parkinsonian Models. Stem Cell Res. Ther..

[132] Zhuo Y., Li W.-S., Lu W., Li X., Ge L.-T., Huang Y., Gao Q.-T., Deng Y.-J., Jiang X.-C., Lan Z.-W. (2024). TGF-Β1 Mediates Hypoxia-Preconditioned Olfactory Mucosa Mesenchymal Stem Cells Improved Neural Functional Recovery in Parkinson’s Disease Models and Patients. Mil. Med. Res..

[133] Lin Y.-Y., Chuang D.-M., Chi C.-Y., Hung S.-Y. (2024). Intranasal Administration of Mesenchymal Stem Cells Overexpressing FGF21 Demonstrates Therapeutic Potential in Experimental Parkinson’s Disease. Neurotherapeutics.

[134] Behzadifard M., Aboutaleb N., Dolatshahi M., Khorramizadeh M., Mirshekari Jahangiri H., Kord Z., Nazarinia D. (2023). Neuroprotective Effects of Conditioned Medium of Mesenchymal Stem Cells (MSC-CM) as a Therapy for Ischemic Stroke Recovery: A Systematic Review. Neurochem. Res..

[135] Xin H., Li Y., Liu Z., Wang X., Shang X., Cui Y., Zhang Z.G., Chopp M. (2013). MiR-133b Promotes Neural Plasticity and Functional Recovery After Treatment of Stroke with Multipotent Mesenchymal Stromal Cells in Rats Via Transfer of Exosome-Enriched Extracellular Particles. Stem Cells.

[136] Zhang H., Wang Y., Lv Q., Gao J., Hu L., He Z. (2018). MicroRNA-21 Overexpression Promotes the Neuroprotective Efficacy of Mesenchymal Stem Cells for Treatment of Intracerebral Hemorrhage. Front. Neurol..

[137] Razavi-Toosi S., Asadi Y., Aboutaleb N., Faezi M. (2023). Conditioned Medium Derived from the Human Amniotic Membrane Prevents Brain Damage Against Cerebral Ischemia/Reperfusion in Subacute, Acute, and Chronic Phases in a Rat Model of Stroke. Basic Clin. Neurosci. J..

[138] Redondo-Castro E., Cunningham C., Miller J., Martuscelli L., Aoulad-Ali S., Rothwell N.J., Kielty C.M., Allan S.M., Pinteaux E. (2017). Interleukin-1 Primes Human Mesenchymal Stem Cells towards an Anti-Inflammatory and pro-Trophic Phenotype In Vitro. Stem Cell Res. Ther..

[139] Wong R., Smith C.J., Allan S.M., Pinteaux E. (2023). Preconditioning with Interleukin-1 Alpha Is Required for the Neuroprotective Properties of Mesenchymal Stem Cells after Ischemic Stroke in Mice. J. Cereb. Blood Flow. Metab..

[140] Cunningham C.J., Wong R., Barrington J., Tamburrano S., Pinteaux E., Allan S.M. (2020). Systemic Conditioned Medium Treatment from Interleukin-1 Primed Mesenchymal Stem Cells Promotes Recovery after Stroke. Stem Cell Res. Ther..

[141] Jashire Nezhad N., Safari A., Namavar M.R., Nami M., Karimi-Haghighi S., Pandamooz S., Dianatpour M., Azarpira N., Khodabandeh Z., Zare S. (2023). Short-Term Beneficial Effects of Human Dental Pulp Stem Cells and Their Secretome in a Rat Model of Mild Ischemic Stroke. J. Stroke Cerebrovasc. Dis..

[142] Yang L.-Y., Chen Y.-R., Lee J.-E., Chen K.-W., Luh H.-T., Chen Y.-T., Wang K.-C., Hsieh S.-T. (2023). Dental Pulp Stem Cell-Derived Conditioned Medium Alleviates Subarachnoid Hemorrhage-Induced Microcirculation Impairment by Promoting M2 Microglia Polarization and Reducing Astrocyte Swelling. Transl. Stroke Res..

[143] Hur H.-J., Lee J.Y., Kim D.-H., Cho M.S., Lee S., Kim H.-S., Kim D.-W. (2022). Conditioned Medium of Human Pluripotent Stem Cell-Derived Neural Precursor Cells Exerts Neurorestorative Effects against Ischemic Stroke Model. Int. J. Mol. Sci..

[144] Wang B., Chen P., Li W., Chen Z. (2024). Exosomes in Stroke Management: A Promising Paradigm Shift in Stroke Therapy. Neural Regen. Res..

[145] Zhang K., Cheng K. (2023). Stem Cell-Derived Exosome versus Stem Cell Therapy. Nat. Rev. Bioeng..

[146] Liang Y., Duan L., Lu J., Xia J. (2021). Engineering Exosomes for Targeted Drug Delivery. Theranostics.

[147] Jiang L., Dong H., Cao H., Ji X., Luan S., Liu J. (2019). Exosomes in Pathogenesis, Diagnosis, and Treatment of Alzheimer’s Disease. Med. Sci. Monit..

[148] Hou B.-R., Jiang C., Wang Z.-N., Ren H.-J. (2020). Exosome-Mediated Crosstalk between Microglia and Neural Stem Cells in the Repair of Brain Injury. Neural Regen. Res..

[149] Perez-Gonzalez R., Gauthier S.A., Kumar A., Levy E. (2012). The Exosome Secretory Pathway Transports Amyloid Precursor Protein Carboxyl-Terminal Fragments from the Cell into the Brain Extracellular Space. J. Biol. Chem..

[150] Rajendran L., Honsho M., Zahn T.R., Keller P., Geiger K.D., Verkade P., Simons K. (2006). Alzheimer’s Disease β-Amyloid Peptides Are Released in Association with Exosomes. Proc. Natl. Acad. Sci. USA.

[151] Picca A., Guerra F., Calvani R., Coelho-Junior H., Bucci C., Marzetti E. (2022). Circulating Extracellular Vesicles: Friends and Foes in Neurodegeneration. Neural Regen. Res..

[152] Reza-Zaldivar E., Hernández-Sapiéns M., Gutiérrez-Mercado Y., Sandoval-Ávila S., Gomez-Pinedo U., Márquez-Aguirre A., Vázquez-Méndez E., Padilla-Camberos E., Canales-Aguirre A. (2019). Mesenchymal Stem Cell-Derived Exosomes Promote Neurogenesis and Cognitive Function Recovery in a Mouse Model of Alzheimer’s Disease. Neural Regen. Res..

[153] Reza-Zaldivar E.E., Hernández-Sapiéns M.A., Minjarez B., Gutiérrez-Mercado Y.K., Márquez-Aguirre A.L., Canales-Aguirre A.A. (2018). Potential Effects of MSC-Derived Exosomes in Neuroplasticity in Alzheimer’s Disease. Front. Cell Neurosci..

[154] Khan M.I., Jeong E.S., Khan M.Z., Shin J.H., Kim J.D. (2023). Stem Cells-Derived Exosomes Alleviate Neurodegeneration and Alzheimer’s Pathogenesis by Ameliorating Neuroinflamation, and Regulating the Associated Molecular Pathways. Sci. Rep..

[155] Alvarez-Erviti L., Seow Y., Yin H., Betts C., Lakhal S., Wood M.J.A. (2011). Delivery of SiRNA to the Mouse Brain by Systemic Injection of Targeted Exosomes. Nat. Biotechnol..

[156] Ma X., Huang M., Zheng M., Dai C., Song Q., Zhang Q., Li Q., Gu X., Chen H., Jiang G. (2020). ADSCs-Derived Extracellular Vesicles Alleviate Neuronal Damage, Promote Neurogenesis and Rescue Memory Loss in Mice with Alzheimer’s Disease. J. Control. Release.

[157] Sul J.H., Shin S., Kim H.K., Han J., Kim J., Son S., Lee J., Baek S.H., Cho Y., Lee J. (2024). Dopamine-conjugated Extracellular Vesicles Induce Autophagy in Parkinson’s Disease. J. Extracell. Vesicles.

[158] Wang C.-C., Hu X.-M., Long Y.-F., Huang H.-R., He Y., Xu Z.-R., Qi Z.-Q. (2024). Treatment of Parkinson’s Disease Model with Human Umbilical Cord Mesenchymal Stem Cell-Derived Exosomes Loaded with BDNF. Life Sci..

[159] Ye J., Sun X., Jiang Q., Gui J., Feng S., Qin B., Xie L., Guo A., Dong J., Sang M. (2024). Umbilical Cord Blood-Derived Exosomes Attenuate Dopaminergic Neuron Damage of Parkinson’s Disease Mouse Model. J. Nanobiotechnol..

[160] Chen C., Chang Z.-H., Yao B., Liu X.-Y., Zhang X.-W., Liang J., Wang J.-J., Bao S.-Q., Chen M.-M., Zhu P. (2024). 3D Printing of Interferon γ-Preconditioned NSC-Derived Exosomes/Collagen/Chitosan Biological Scaffolds for Neurological Recovery after TBI. Bioact. Mater..

[161] Gu C., Li Y., Liu J., Liu S., Long J., Zhang Q., Duan W., Feng T., Huang J., Qiu Y. (2023). Neural Stem Cell-Derived Exosomes-Loaded Adhesive Hydrogel Controlled-Release Promotes Cerebral Angiogenesis and Neurological Function in Ischemic Stroke. Exp. Neurol..

[162] Zhang Q., Liu T., Li Y., Fan Y., Shang H., Zhao H., Sun H., Yu Z., Han M., Wan C. (2024). Gelatin Methacryloyl Microneedle Loaded with 3D-MSC-Exosomes for the Protection of Ischemia-Reperfusion. Int. J. Biol. Macromol..

[163] Han M., Zhang Z., Liu Z., Liu Y., Zhao H., Wang B., Zhang C., Shang H., Li Y., Wang S. (2023). Three-Dimensional-Cultured MSC-Derived Exosome with Hydrogel for Cerebral Ischemia Repair. Biomater. Adv..

[164] Kang H., Huang Y., Peng H., Zhang X., Liu Y., Liu Y., Xia Y., Liu S., Wu Y., Wang S. (2024). Mesenchymal Stem Cell-Loaded Hydrogel Improves Surgical Treatment for Chronic Cerebral Ischemia. Transl. Stroke Res..

[165] Cakir B., Kiral F.R., Park I.-H. (2022). Advanced in Vitro Models: Microglia in Action. Neuron.

[166] Galatro T.F., Vainchtein I.D., Brouwer N., Boddeke E.W.G.M., Eggen B.J.L. (2017). Isolation of Microglia and Immune Infiltrates from Mouse and Primate Central Nervous System. Inflammation. Methods in Molecular Biology.

[167] Gosselin D., Skola D., Coufal N.G., Holtman I.R., Schlachetzki J.C.M., Sajti E., Jaeger B.N., O’Connor C., Fitzpatrick C., Pasillas M.P. (2017). An Environment-Dependent Transcriptional Network Specifies Human Microglia Identity. Science.

[168] Burns T.C., Li M.D., Mehta S., Awad A.J., Morgan A.A. (2015). Mouse Models Rarely Mimic the Transcriptome of Human Neurodegenerative Diseases: A Systematic Bioinformatics-Based Critique of Preclinical Models. Eur. J. Pharmacol..

[169] Dolan M.-J., Therrien M., Jereb S., Kamath T., Gazestani V., Atkeson T., Marsh S.E., Goeva A., Lojek N.M., Murphy S. (2023). Exposure of IPSC-Derived Human Microglia to Brain Substrates Enables the Generation and Manipulation of Diverse Transcriptional States In Vitro. Nat. Immunol..

[170] Muffat J., Li Y., Yuan B., Mitalipova M., Omer A., Corcoran S., Bakiasi G., Tsai L.-H., Aubourg P., Ransohoff R.M. (2016). Efficient Derivation of Microglia-like Cells from Human Pluripotent Stem Cells. Nat. Med..

[171] Douvaras P., Sun B., Wang M., Kruglikov I., Lallos G., Zimmer M., Terrenoire C., Zhang B., Gandy S., Schadt E. (2017). Directed Differentiation of Human Pluripotent Stem Cells to Microglia. Stem Cell Rep..

[172] Abud E.M., Ramirez R.N., Martinez E.S., Healy L.M., Nguyen C.H.H., Newman S.A., Yeromin A.V., Scarfone V.M., Marsh S.E., Fimbres C. (2017). IPSC-Derived Human Microglia-like Cells to Study Neurological Diseases. Neuron.

[173] Banerjee P., Paza E., Perkins E.M., James O.G., Kenkhuis B., Lloyd A.F., Burr K., Story D., Yusuf D., He X. (2020). Generation of Pure Monocultures of Human Microglia-like Cells from Induced Pluripotent Stem Cells. Stem Cell Res..

[174] Brownjohn P.W., Smith J., Solanki R., Lohmann E., Houlden H., Hardy J., Dietmann S., Livesey F.J. (2018). Functional Studies of Missense TREM2 Mutations in Human Stem Cell-Derived Microglia. Stem Cell Rep..

[175] Chen Y., Colonna M. (2021). Microglia in Alzheimer’s Disease at Single-Cell Level. Are There Common Patterns in Humans and Mice?. J. Exp. Med..

[176] Grubman A., Choo X.Y., Chew G., Ouyang J.F., Sun G., Croft N.P., Rossello F.J., Simmons R., Buckberry S., Landin D.V. (2021). Transcriptional Signature in Microglia Associated with Aβ Plaque Phagocytosis. Nat. Commun..

[177] Guttikonda S.R., Sikkema L., Tchieu J., Saurat N., Walsh R.M., Harschnitz O., Ciceri G., Sneeboer M., Mazutis L., Setty M. (2021). Fully Defined Human Pluripotent Stem Cell-Derived Microglia and Tri-Culture System Model C3 Production in Alzheimer’s Disease. Nat. Neurosci..

[178] Haenseler W., Sansom S.N., Buchrieser J., Newey S.E., Moore C.S., Nicholls F.J., Chintawar S., Schnell C., Antel J.P., Allen N.D. (2017). A Highly Efficient Human Pluripotent Stem Cell Microglia Model Displays a Neuronal-Co-Culture-Specific Expression Profile and Inflammatory Response. Stem Cell Rep..

[179] Konttinen H., Cabral-da-Silva M.e.C., Ohtonen S., Wojciechowski S., Shakirzyanova A., Caligola S., Giugno R., Ishchenko Y., Hernández D., Fazaludeen M.F. (2019). PSEN1ΔE9, APPswe, and APOE4 Confer Disparate Phenotypes in Human IPSC-Derived Microglia. Stem Cell Rep..

[180] Takata K., Kozaki T., Lee C.Z.W., Thion M.S., Otsuka M., Lim S., Utami K.H., Fidan K., Park D.S., Malleret B. (2017). Induced-Pluripotent-Stem-Cell-Derived Primitive Macrophages Provide a Platform for Modeling Tissue-Resident Macrophage Differentiation and Function. Immunity.

[181] Pandya H., Shen M.J., Ichikawa D.M., Sedlock A.B., Choi Y., Johnson K.R., Kim G., Brown M.A., Elkahloun A.G., Maric D. (2017). Differentiation of Human and Murine Induced Pluripotent Stem Cells to Microglia-like Cells. Nat. Neurosci..

[182] Ihnatovych I., Birkaya B., Notari E., Szigeti K. (2020). IPSC-Derived Microglia for Modeling Human-Specific DAMP and PAMP Responses in the Context of Alzheimer’s Disease. Int. J. Mol. Sci..

[183] Rostami J., Mothes T., Kolahdouzan M., Eriksson O., Moslem M., Bergström J., Ingelsson M., O’Callaghan P., Healy L.M., Falk A. (2021). Crosstalk between Astrocytes and Microglia Results in Increased Degradation of α-Synuclein and Amyloid-β Aggregates. J. Neuroinflamm..

[184] Trudler D., Nazor K.L., Eisele Y.S., Grabauskas T., Dolatabadi N., Parker J., Sultan A., Zhong Z., Goodwin M.S., Levites Y. (2021). Soluble α-Synuclein–Antibody Complexes Activate the NLRP3 Inflammasome in HiPSC-Derived Microglia. Proc. Natl. Acad. Sci. USA.

[185] Fontainhas A.M., Wang M., Liang K.J., Chen S., Mettu P., Damani M., Fariss R.N., Li W., Wong W.T. (2011). Microglial Morphology and Dynamic Behavior Is Regulated by Ionotropic Glutamatergic and GABAergic Neurotransmission. PLoS ONE.

[186] Stogsdill J.A., Kim K., Binan L., Farhi S.L., Levin J.Z., Arlotta P. (2022). Pyramidal Neuron Subtype Diversity Governs Microglia States in the Neocortex. Nature.

[187] Szepesi Z., Manouchehrian O., Bachiller S., Deierborg T. (2018). Bidirectional Microglia–Neuron Communication in Health and Disease. Front. Cell Neurosci..

[188] Stöberl N., Maguire E., Salis E., Shaw B., Hall-Roberts H. (2023). Human IPSC-Derived Glia Models for the Study of Neuroinflammation. J. Neuroinflamm..

[189] Bassil R., Shields K., Granger K., Zein I., Ng S., Chih B. (2021). Improved Modeling of Human AD with an Automated Culturing Platform for IPSC Neurons, Astrocytes and Microglia. Nat. Commun..

[190] Choi J., Choi H.K., Lee K. (2023). In Situ Detection of Neuroinflammation Using Multicellular 3D Neurovascular-Unit-on-a-Chip. Adv. Funct. Mater..

[191] Pediaditakis I., Kodella K.R., Manatakis D.V., Le C.Y., Barthakur S., Sorets A., Gravanis A., Ewart L., Rubin L.L., Manolakos E.S. (2022). A Microengineered Brain-Chip to Model Neuroinflammation in Humans. iScience.

[192] Schwartz M.P., Hou Z., Propson N.E., Zhang J., Engstrom C.J., Costa V.S., Jiang P., Nguyen B.K., Bolin J.M., Daly W. (2015). Human Pluripotent Stem Cell-Derived Neural Constructs for Predicting Neural Toxicity. Proc. Natl. Acad. Sci. USA.

[193] Berjaoui C., Kachouh C., Joumaa S., Hussein Ghayyad M., Abate Bekele B., Rita A., Al Maaz Z., Awde S., Wojtara M., Nazir A. (2024). Neuroinflammation-on-a-Chip for Multiple Sclerosis Research: A Narrative Review. Ann. Med. Surg..

[194] Amartumur S., Nguyen H., Huynh T., Kim T.S., Woo R.-S., Oh E., Kim K.K., Lee L.P., Heo C. (2024). Neuropathogenesis-on-Chips for Neurodegenerative Diseases. Nat. Commun..

[195] Sabate-Soler S., Kurniawan H., Schwamborn J.C. (2024). Advanced Brain Organoids for Neuroinflammation Disease Modeling. Neural Regen. Res..

[196] Lancaster M.A., Knoblich J.A. (2014). Generation of Cerebral Organoids from Human Pluripotent Stem Cells. Nat. Protoc..

[197] Lancaster M.A., Renner M., Martin C.-A., Wenzel D., Bicknell L.S., Hurles M.E., Homfray T., Penninger J.M., Jackson A.P., Knoblich J.A. (2013). Cerebral Organoids Model Human Brain Development and Microcephaly. Nature.

[198] Velasco S., Kedaigle A.J., Simmons S.K., Nash A., Rocha M., Quadrato G., Paulsen B., Nguyen L., Adiconis X., Regev A. (2019). Individual Brain Organoids Reproducibly Form Cell Diversity of the Human Cerebral Cortex. Nature.

[199] Eigenhuis K.N., Somsen H.B., van der Kroeg M., Smeenk H., Korporaal A.L., Kushner S.A., de Vrij F.M.S., van den Berg D.L.C. (2023). A Simplified Protocol for the Generation of Cortical Brain Organoids. Front. Cell Neurosci..

[200] Paşca A.M., Sloan S.A., Clarke L.E., Tian Y., Makinson C.D., Huber N., Kim C.H., Park J.-Y., O’Rourke N.A., Nguyen K.D. (2015). Functional Cortical Neurons and Astrocytes from Human Pluripotent Stem Cells in 3D Culture. Nat. Methods.

[201] Miura Y., Li M.-Y., Birey F., Ikeda K., Revah O., Thete M.V., Park J.-Y., Puno A., Lee S.H., Porteus M.H. (2020). Generation of Human Striatal Organoids and Cortico-Striatal Assembloids from Human Pluripotent Stem Cells. Nat. Biotechnol..

[202] Mulder L.A., Depla J.A., Sridhar A., Wolthers K., Pajkrt D., Vieira de Sá R. (2023). A Beginner’s Guide on the Use of Brain Organoids for Neuroscientists: A Systematic Review. Stem Cell Res. Ther..

[203] Zhang W., Jiang J., Xu Z., Yan H., Tang B., Liu C., Chen C., Meng Q. (2023). Microglia-Containing Human Brain Organoids for the Study of Brain Development and Pathology. Mol. Psychiatry.

[204] Chambers S.M., Fasano C.A., Papapetrou E.P., Tomishima M., Sadelain M., Studer L. (2009). Highly Efficient Neural Conversion of Human ES and IPS Cells by Dual Inhibition of SMAD Signaling. Nat. Biotechnol..

[205] Cakir B., Xiang Y., Tanaka Y., Kural M.H., Parent M., Kang Y.-J., Chapeton K., Patterson B., Yuan Y., He C.-S. (2019). Engineering of Human Brain Organoids with a Functional Vascular-like System. Nat. Methods.

[206] Sun X.-Y., Ju X.-C., Li Y., Zeng P.-M., Wu J., Zhou Y.-Y., Shen L.-B., Dong J., Chen Y.-J., Luo Z.-G. (2022). Generation of Vascularized Brain Organoids to Study Neurovascular Interactions. eLife.

[207] Choi S.H., Kim Y.H., Hebisch M., Sliwinski C., Lee S., D’Avanzo C., Chen H., Hooli B., Asselin C., Muffat J. (2014). A Three-Dimensional Human Neural Cell Culture Model of Alzheimer’s Disease. Nature.

[208] Raja W.K., Mungenast A.E., Lin Y.-T., Ko T., Abdurrob F., Seo J., Tsai L.-H. (2016). Self-Organizing 3D Human Neural Tissue Derived from Induced Pluripotent Stem Cells Recapitulate Alzheimer’s Disease Phenotypes. PLoS ONE.

[209] Sreenivasamurthy S., Laul M., Zhao N., Kim T., Zhu D. (2023). Current Progress of Cerebral Organoids for Modeling Alzheimer’s Disease Origins and Mechanisms. Bioeng. Transl. Med..

[210] Fernandes S., Revanna J., Pratt J., Hayes N., Marchetto M.C., Gage F.H. (2024). Modeling Alzheimer’s Disease Using Human Cell Derived Brain Organoids and 3D Models. Front. Neurosci..

[211] Park J.-C., Jang S.-Y., Lee D., Lee J., Kang U., Chang H., Kim H.J., Han S.-H., Seo J., Choi M. (2021). A Logical Network-Based Drug-Screening Platform for Alzheimer’s Disease Representing Pathological Features of Human Brain Organoids. Nat. Commun..

[212] Park J., Wetzel I., Marriott I., Dréau D., D’Avanzo C., Kim D.Y., Tanzi R.E., Cho H. (2018). A 3D Human Triculture System Modeling Neurodegeneration and Neuroinflammation in Alzheimer’s Disease. Nat. Neurosci..

[213] Pomeshchik Y., Klementieva O., Gil J., Martinsson I., Hansen M.G., de Vries T., Sancho-Balsells A., Russ K., Savchenko E., Collin A. (2020). Human IPSC-Derived Hippocampal Spheroids: An Innovative Tool for Stratifying Alzheimer Disease Patient-Specific Cellular Phenotypes and Developing Therapies. Stem Cell Rep..

[214] Cui X., Li X., Zheng H., Su Y., Zhang S., Li M., Hao X., Zhang S., Hu Z., Xia Z. (2024). Human Midbrain Organoids: A Powerful Tool for Advanced Parkinson’s Disease Modeling and Therapy Exploration. NPJ Park. Dis..

[215] Kim H., Park H.J., Choi H., Chang Y., Park H., Shin J., Kim J., Lengner C.J., Lee Y.K., Kim J. (2019). Modeling G2019S-LRRK2 Sporadic Parkinson’s Disease in 3D Midbrain Organoids. Stem Cell Rep..

[216] Smits L.M., Reinhardt L., Reinhardt P., Glatza M., Monzel A.S., Stanslowsky N., Rosato-Siri M.D., Zanon A., Antony P.M., Bellmann J. (2019). Modeling Parkinson’s Disease in Midbrain-like Organoids. NPJ Park. Dis..

[217] Kim M.S., Kim D.-H., Kang H.K., Kook M.G., Choi S.W., Kang K.-S. (2021). Modeling of Hypoxic Brain Injury through 3D Human Neural Organoids. Cells.

[218] Wang S., Wang Z., Wang X., Zhang X., Xu T., Miao C. (2023). Humanized Cerebral Organoids-Based Ischemic Stroke Model for Discovering of Potential Anti-Stroke Agents. Acta Pharmacol. Sin..

